# Nursing consultation and diabetes: an educational and transformative process for primary health care^*^

**DOI:** 10.1590/1518-8345.7546.4464

**Published:** 2025-02-17

**Authors:** Silvana de Oliveira Silva, Andréa Carvalho Araújo Moreira, Alexa Pupiara Flores Coelho Centenaro, Nara Marilene Oliveira Girardon-Perlini, Teresinha Heck Weiller, Maria Denise Schimith

**Affiliations:** 1 Prefeitura Municipal de Santiago, Secretaria Municipal de Saúde, Santiago, Rio Grande do Sul, RS, Brasil.; 2 Universidade Federal de Santa Maria, Santa Maria, Rio Grande do Sul, RS, Brasil.; 3 Universidade Estadual Vale do Acaraú, Sobral, Ceará, CE, Brasil.; 4 Universidade Federal de Santa Maria, Palmeira das Missões, Rio Grande do Sul, RS, Brasil.

**Keywords:** Nursing Process, Diabetes Mellitus, Primary Health Care, Healthcare Models, Chronic Diseases, Problem-Based Learning.

## Abstract

**Objective: to analyze the constituent elements that support nursing consultations with people with diabetes *mellitus*, and to develop an educational process based on the assumptions of the Chronic Conditions Care Model and mediated by a reflective-dialogical process with nurses, aiming to improve the quality of the practice in the Family Health Strategy.:**

**Method:**

convergent care research carried out with 12 nurses. Participant observation, semi-structured individual interviews and convergence groups were used to collect data, which were treated by participatory analysis, with an interpretative approach.

**Results:**

the promotion of self-care, the bond between professionals and users, and the support for lifestyle changes for people with diabetes were aligned with the proposed Care Model. On the other hand, attitudes, values and knowledge that weaken care were identified as divergent. The theoretical and practical deepening of the care model, diabetes, dealing with work overload, and the implementation of a guide and a protocol for the development of the nursing consultation were points of convergence for improving the quality of the consultation. It was also observed that nurses’ autonomy was strengthened, critical thinking was awakened, and the search for improvement and redefinition of the relationship with the user was sought.

**Conclusion:**

the nursing consultation was enhanced through the active participation of nurses in an educational, reflective and dialogical process.

## Highlights

**(1)** The Nursing Consultation for people with DM by nurses consisted of elements that are similar to and different from the MACC. **(2)** Elements that align with the MACC: as establishing a bond with users, accountability, longitudinal care, and the social and family approach. **(3)** Elements diverged from the assumptions of MACC: a limited understanding of the care model, the professional attitude of blaming the user and the weaknesses in the application of the nursing process. **(4)** The nursing consultation was enhanced through the active participation of nurses in an educational, reflective and dialogical process.

## Introduction

Diabetes *mellitus* (DM) is a chronic non-communicable disease that caused approximately 2.0 million deaths worldwide in 2019. From 2000 to 2019, DM recorded a global increase of 3% in the number of deaths, and in the Americas, it surpassed stroke in terms of years of life adjusted for disability, thus ranking second^1^.

According to the International Diabetes Federation (IDF), the prevalence of the disease in low-income countries is 5.5%^2^. In Brazil, an incidence of 8.6% was identified, with 68.2% of the population being aware of their diagnosis, 92.2% of those who are aware of their diagnosis undergoing drug treatment, and of these, only 35.8% have controlled glycated hemoglobin levels^3^. 

Therefore, it is essential to pay attention to the current and future damage caused by this disease, in order to implement effective public policies, as most countries have made little progress in meeting Sustainable Development Goal 3.4, which foresees a one-third reduction in premature mortality from non-communicable diseases between 2015 and 2030. In this context, screening and treatment of DM are high-priority interventions to reduce mortality from chronic diseases^4^. 

Primary Health Care (PHC) in Brazil, organized at the national level through the Family Health Strategy (FHS), should be the preferred entry point for people who have DM into the health system. It is the strategic point of care to better accommodate health needs, including longitudinal and continuous monitoring^5^. Treatments for DM are based on non-pharmacological and pharmacological approaches to glycemic control, which must be chosen considering the biopsychosocial-spiritual aspects of each person, through an interpersonal relationship between professional and patient, because this directly influences adherence to treatment.

Hence, the nurse is the professional in first contact with the patient, who must assume a leadership role in the detection, treatment and rehabilitation of diabetes. It is also up to this professional to support efforts to prevent the disease and to promote the health of people living with diabetes, thus allowing them to participate in decision-making related to their treatment^6^. 

For the World Health Organization (WHO), the contribution of nursing to preventing and managing diabetes in the health system is demonstrated by six characteristics: 1) coordination of care to ensure that patients’ health needs are met over time; 2) participation in a multidisciplinary approach to care based on an integrated relationship between health professionals, allowing different professionals to work as a team to improve the quality of care; 3) mobilization and training of the nursing workforce to specialize in noncommunicable diseases, aiming for more effective and sustainable treatments; 4) improvement of access to care; 5) empowerment of individuals and the community; and 6) utilization of technology to maintain access to essential health services and reduce exposure to COVID-19^7^.

There are efforts by Brazilian nurses to contribute to adherence to DM treatment, providing scientific bases for nursing care, with the aim of directing actions that promote greater adherence to treatment with the active participation of the patient, reducing complications, hospitalizations and mortality^8^. On the other hand, an international study^9^ showed that diabetes is still not a priority in primary health centers. The nurses explained the importance of working on primary prevention of diabetes, prioritizing the care of people with pre-diabetes, and pointed out the need to establish clinical guidelines, support in care knowledge and skills, in addition to the implementation of an interprofessional work process, crucial aspects in the health care of people with diabetes that are based on the Chronic Conditions Care Model (MACC, in Portuguese). 

The MACC was proposed by Eugênio Vilaça Mendes to meet the specific needs of the Brazilian public health system. It has its origins in the Chronic Care Model (CCM) from the United States, and incorporates two other models, the Risk Pyramid Model (RPM) and the Social Determinants of Health Model, to adapt to the requirements of a public and universal health care system such as the *Sistema Único de Saúde* (SUS)^10^. This is an operational instrument, established in the National Plan to Combat Chronic Conditions to achieve the millennium development goals by 2030.

The nursing consultation (NC) guides the nurse’s critical thinking and clinical judgment, directing it towards the care of the person, family, community and special groups. In the context of the FHS and care for people with chronic conditions, the NC favors resolute, proactive and humanized care, based on educational actions capable of empowering users to achieve goals established in the care and self-care process.

However, the difficulties faced in the practice of NC within the scope of PHC are worrying, such as: lack of time, lack of agility for nursing diagnosis, problems in the interface between care and management, and excessive bureaucratic demands^11^.

In the scientific field, there is much to be investigated about nursing consultation for people with diabetes in the context of PHC, given its relevance, especially when associated with the MACC assumptions. There are few studies on this topic, which is committed to promoting changes and/or innovations in healthcare practice, adopting a constant perspective of constructing thinking and doing.

In view of the above, this research aims to analyze the constituent elements (knowledge, technologies, beliefs and values) that support nursing consultation with people with diabetes *mellitus*, and to develop an educational process, based on the assumptions of the Chronic Conditions Care Model and mediated by a reflective-dialogical process with nurses, aiming to improve the quality of the practice in the Family Health Strategy.

## Method

### Study design

Convergent care research (CCR) that aims, during the research process, to promote changes and/or innovations in care practice, based on the continuous construction of thinking and doing^12^. Its development adhered to the recommendations of the Consolidated Criteria for Reporting Qualitative Research^13^. 

### Study location

The study was carried out in a medium-sized city located in the central-west region of the state of Rio Grande do Sul, Brazil, which is part of the Health Care Planning project and has 11 FHS units, covering 86% of the total population.

### Period

The data was produced between August 2020 and December 2021, during which the COVID-19 pandemic was ravaging the country. All precautions to prevent transmission were taken. 

### Participants and selection criteria

Nurses who worked in the FHS and had at least 6 months of experience in NC with people who have DM were included [according to Article 442-A of the Brazilian Consolidation of Labor Laws (CLT, in Portuguese), this is the maximum probationary period that employers can require for hiring purposes]. The exclusion criterion was established as follows: nurses who for some reason were away from work for more than 30 days during the data collection period, which did not occur. Thus, 12 nurses were contacted by email for a meeting to present the proposal and, in all cases, they agreed to participate in the study. 

### Data collection

Triangulation of techniques was adopted for data production: active participant observation^14^, semi-structured interviews^15^ and convergence groups^16^. 

The objective of participant observation was to capture and record the most varied elements and impressions about the conduct of NC for people who have DM. Therefore, a pre-established script was used, which included the following aspects: professional relationship with the user, communication, stages of the nursing process, nursing record, and environment.

The researcher’s interaction during the observations occurred gradually. During the NC, she sought to maintain a more passive position, interacting strictly when necessary. Active participation occurred mainly after the end of the consultation, when, between one appointment and another, there was a dialogue about the case and knowledge was shared based on the researcher’s perceptions and the doubts listed by the participants.

The interruption of observations was established using the theoretical saturation criterion^17^, which consisted of individual compilation, seeking to identify whether the elements proposed by the script were explored. Once all elements had been covered, and no other new information relevant to the study topic emerged, observations were concluded. Totaling 120 hours, and with an average of 10 hours/participant, the data produced at this stage were recorded in a field diary and digital recorder, immediately after completion, coded and later transcribed into the Microsoft Word text editor.

The semi-structured individual interview was carried out by the researcher after reaching observation saturation and before the convergence groups. To this end, they were encouraged to talk about the daily routine of CE with people who have DM, addressing aspects such as objective, what they do and how they do it; about the MACC, their knowledge and applicability in daily life; about the challenges and potential for carrying out CE with people who have DM; and suggestions for the convergence groups. A pilot test was applied, but was not part of the sample of participants. Twelve interviews were conducted, previously scheduled with the participants, in the health units, audio-recorded, with an average duration of 40 minutes. Then, a trained research assistant transcribed the material in the *Microsoft Word* text editor.

The data obtained through these two techniques supported the Convergence Group (CG), which aims to carry out research simultaneously with healthcare practice within the collective, focusing on health education or clinical practice. In this space, discussions are promoted on a specific topic, the focus of the research, in an interactive process between participants and researchers, in order to allow dialogicity^18^. 

The meetings were held according to a schedule developed with the participants, in the meeting room of the Municipal Health Department, with an average duration of 2 hours and 30 minutes. They were audio-recorded using a digital recorder, and the researcher was assisted by a research assistant, a student in the Master’s Program in Nursing, who received training and actively contributed to the planning, execution, observation and transcription. 

To achieve cohesion, the convergence group went through a four-phase process, called the Four Rs Process: recognition, revelation, redistribution and rethinking^16^. The purpose of the recognition phase was to enable the group’s first meeting and establish relationships between participants and the researcher so that group cohesion could occur through participatory dialogue. This phase consisted of two meetings. The first aimed to analyze participants’ knowledge about the NC in terms of its purpose and assumptions. To this end, group activities were carried out, including the creation of posters, dramatization and debates. The second meeting focused on knowledge about the MACC, using keywords and images as resources to promote interaction and dialogue.

The revelation phase comprised the moment in which participants identified with their peers through their shared experience with the proposal of the meetings. This process was triggered when they were encouraged to talk about their experiences with NC with people who have DM and to develop the concepts that guide their care practice. 

The redistribution phase was the moment in which the group, mediated by the exchange of experiences, was led to make shared decisions regarding the purpose of the meeting. This phase had three meetings and included a more active participation by the researcher. The activities were supported by reading, discussion of scientific materials, dramatizations, case studies and matrix support from a nutritionist.

The rethinking phase was characterized by the group’s reflection on the implications of the problems identified and the possibility of transferring what was shared and learned to the participants’ daily lives^16^. This phase took place in three moments, with the use of strategies such as: Brainstorming technique, case studies, group dynamics and preparation of the guide for NC with people who have DM.

The group meetings were planned and developed taking as a reference Paulo Freire’s Liberating Pedagogy, which has been used in studies in the areas of education, health and nursing. At this point, Convergent Care Research is linked to this framework, since it is grounded on liberating critical dialogue, based on the principle that critical reflection leads to action (practice), called praxis^19^.

### Data analysis

The analysis was based on participatory research with an interpretative approach, carried out through the construction and feedback of narratives^20^. Built on the assumptions of Gadamer’s Hermeneutic Theory, this approach considers that acts of interpretation are dialogical, exploring participatory strategies and suggesting some changes in the classic way of conducting research. Among these changes, two strategies are used: narrative construction and validation, and participatory consensus production^20^. In this way, the participant engages in the investigation process, which enables the resignification and self-reflection of their actions^21^. 

During the process of transcribing and organizing the data, several in-depth readings were carried out with the aim of understanding and exploring new testimonies. Thus, it was possible to perceive recurring, peculiar, divergent reports and expressed contradictions involving the practice of NC, and to make the first inferences and questions. 

Given these considerations, the analysis was carried out as follows: after transcribing each of the semi-structured interviews (n=12), a narrative was constructed. Afterwards, the researcher returned to the field and read the narrative to each participant and, through the dialogic process, the participant reflected on the narrative and had the opportunity to contest or reinforce and validate it. With the convergence groups, in the same way, nine narratives were produced, one from each meeting, which were read and validated with the participants as the meetings took place. Before the next group began, the possibility of co-production by the participants was reinforced.

In order to facilitate the organization of this data and the categorization of the argumentative cores, the observation notes and the narratives of the interviews and groups were inserted into NVivo Corporate Software. Therefore, with the development of 21 narratives and observation notes, it was possible to construct and link the argumentative cores, which sought to answer the research question^20^.

### Ethical aspects

The research followed the guidelines of Resolution nº 466/12 of the National Health Council^22^, as well as Resolution nº 510/2016 of the Ministry of Health, which emphasizes the ethical specificities of research in the human and social sciences and others that use methodologies specific to these fields^23^. It was approved by the Research Ethics Committee of the *Universidade Federal de Santa Maria*, under opinion number nº 4.209.003. All participants agreed to participate in the study and signed the Free and Informed Consent Form. Anonymity is guaranteed with the following codes: O (Observation) followed by the letter E (nurse), numerals 1 to 12 and date (day/month/year); NE (Nurse Narrative) followed by numerals 1 to 12; NGC (Convergence Group Narrative) followed by numerals 1 to 9. 

## Results

Twelve nurses participated in the study, mostly women (n=11), with an average age of 42 years (minimum of 33 years and maximum of 51 years). The average time since graduation in Nursing was 12 years (minimum of 5 years and maximum of 25 years), and the time working in the FHS was, on average, 7.5 years, with the shortest time being 6 months (n=1) and the longest 11 years (n=3).

The results will be presented through the following argumentative cores: Constituent elements of Nursing Consultation with people who have DM that align with and diverge from the Chronic Conditions Care Model assumptions; and Now it makes sense: the convergence between research and the educational process.

### Constituent elements of Nursing Consultation with people who have DM that align with and diverge from the MACC assumptions

**Figure 1:**
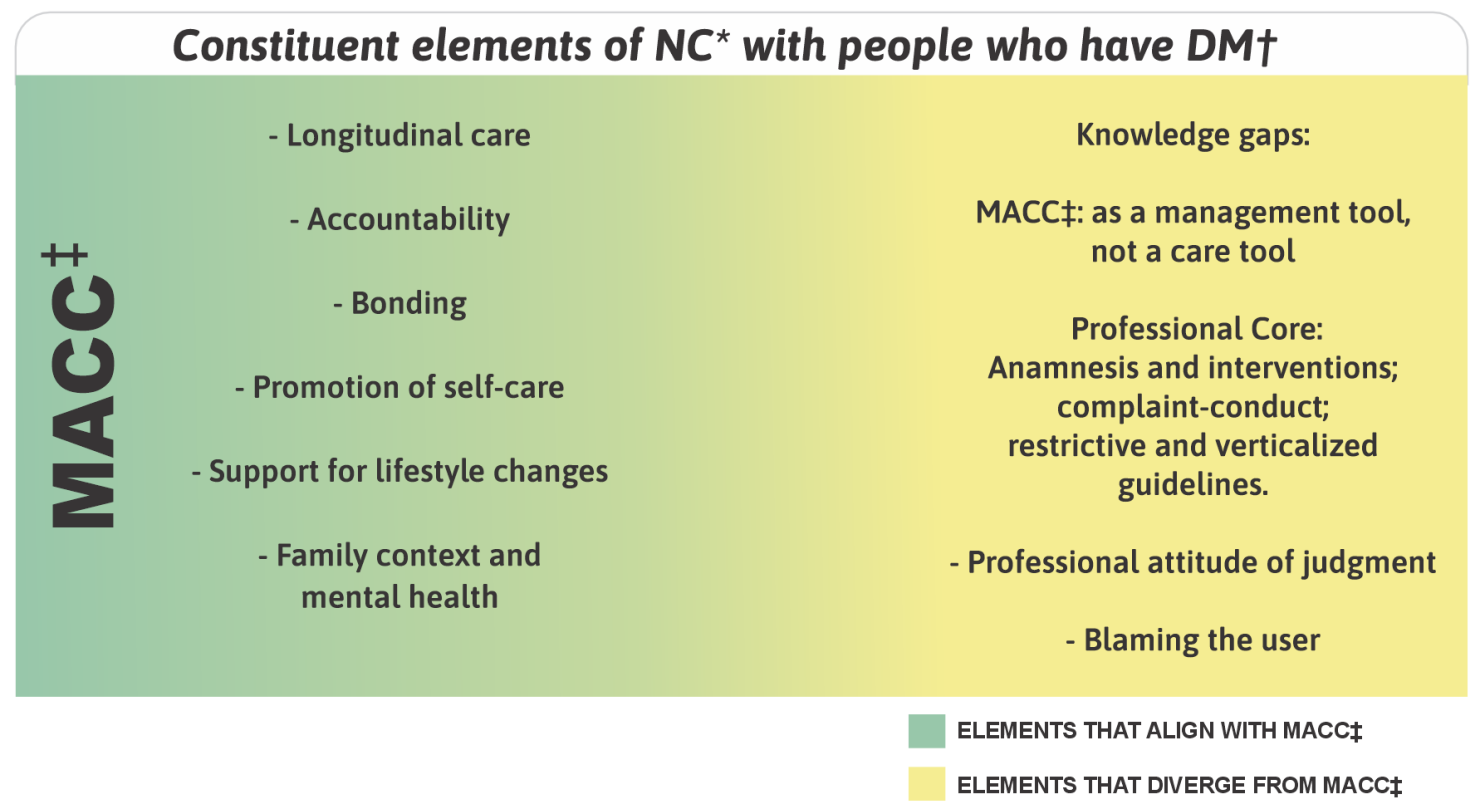
Constituent elements of Nursing Consultation with people who have diabetes *mellitus*

The elements of the NC with people who have diabetes in the FHS include characteristics that align with and diverge from the MACC, as demonstrated in Figure 1.

*NC = Nursing consultation; ^†^DM = Diabetes *mellitus*; ^‡^MACC = Chronic Conditions Care Model

Nurses face some challenges in carrying out CE with people with diabetes that have persisted for some years, namely: work overload, lack of nursing protocols and standard operating procedures (SOPs), and the insufficient number of nursing professionals to meet care and management demands. [...] *I believe that what most limits user assistance during consultations is overload! I am here to provide assistance to users, but that is not all I do! There are management issues related to the service, as well as spontaneous and administrative demands* [...] (NE3). [...] *the nurse was interrupted several times during the consultation to resolve administrative issues related to the service, such as requesting materials, receiving supplies, contacting another service, informing the vacation period of the community agent; she asked for permission and left the room to assess an injury in the dressing room* (NO 08/09/2020)*. We recognize the need to develop and implement SOPs and nursing protocols, because we witness situations every day in which, if we had these instruments, we would have more autonomy* [...] *not to mention better quality in patient care. This way, our time would be better used* [...]. *For these reasons, looking for the issue of resolution in primary care, we understand that one of the possibilities are protocols, the SOPs.* (NGC1). *Even before the pandemic, the number of nursing professionals was already insufficient. We were no longer able to cope because we would actually need one nurse for the care area and another for management. And we only have two nursing technicians on the team; however, there is often one who is unavailable due to being on vacation, on duty, on leave, off, or engaged in another activity, such as now, when one is assigned to another unit to provide support for vaccination.* (NE10).

On the other hand, the participants recognized some contextual factors as means of enhancing nursing consultation in the FHS for people with diabetes, such as support from municipal management, the city’s health care network, and sharing of care with the specialty outpatient clinic; the population’s recognition of the professional performance of nurses; and the bond with users and the professional relationship with the unit’s doctor: [...] *I feel included in this management. Our demands are met; we have protected hours for team meetings and training* (NE9). *What enhances the consultation is that primary care is very strong in the city.* [...] *Users have access, support, recognize the unit as a point of entry, and acknowledge our work as nurses* [...] (NE1). *The issue of interconsultations with the doctor, as it helps in resolving it* (NE4). [...] *we have a specialized outpatient clinic to refer users who need evaluation* [...] *and they always give us feedback to implement care* (NGC3). [...] *we also have a health care network for users here in the city, such as laboratories, a social assistance reference center, a family health support center, nutrition and we can make all of this available to them at the Consultation, the user has access whenever they need it.* (NE7).

### Now it makes sense: the convergence between research and the educational process 

**Figure 2:**
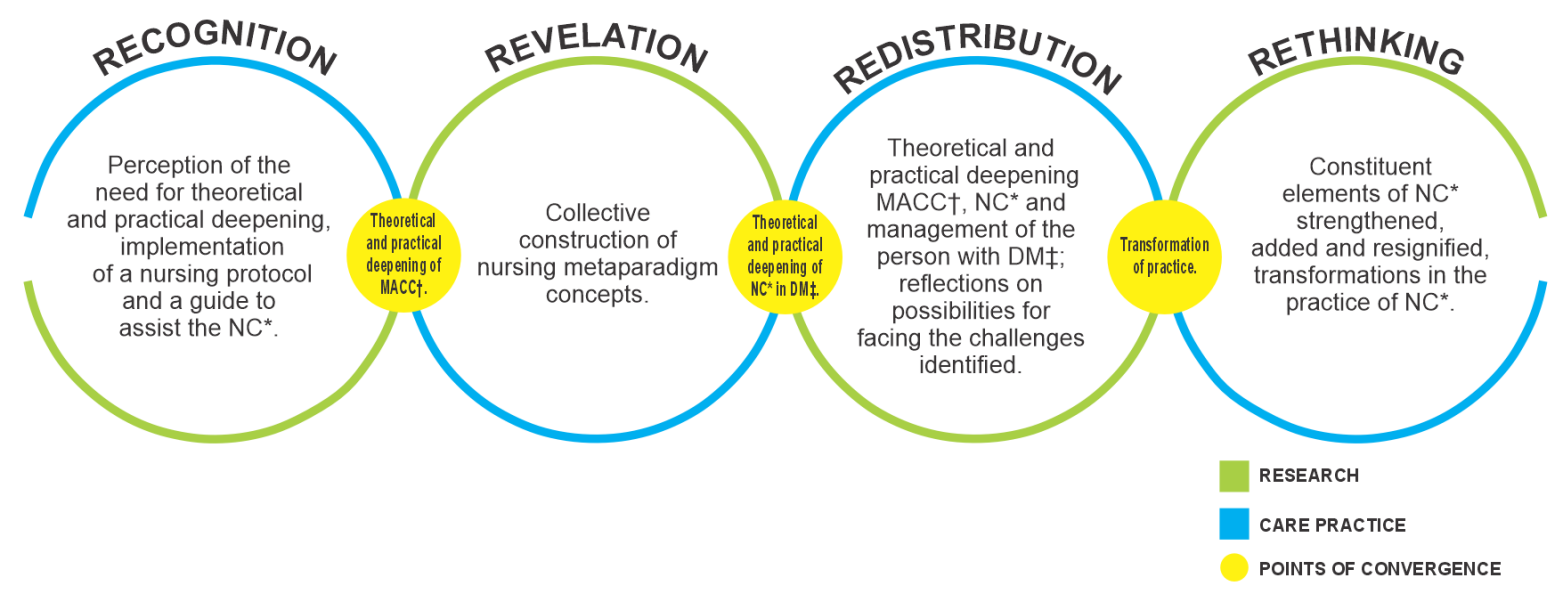
Points of convergence between research and healthcare practice

From the convergence between research and care practice, demands emerged to qualify the Nursing Consultation in the FHS with people who have DM. The educational process developed in the convergence groups is represented in Figure 2.

*NC = Nursing consultation; ^†^MACC = Chronic Conditions Care Model; ^‡^DM = Diabetes *mellitus*

In this study, the exchange of knowledge between the researcher and participants supports concrete actions for change and reflection-action movements that resulted in the collective construction of a guide for NC with people who have DM and the implementation of a nursing protocol. These changes are represented in Figure 3.

**Figure 3:** Transformations in the daily routine of Nursing Consultation based on the educational-dialogical process

Perception of the need for theoretical and practical deepening of NC*	[...] *We need to deepen our knowledge about DM* ^*†*^ and user care. Establish a nursing protocol and a NC* guide to help us in our daily work and better structure the NC*, the entire process, data collection and interventions in theoretical frameworks (NGC^‡^7). [...] *In the planning workshops, the way in which the MACC* ^*§*^ was presented was denser, with the focus on organizing the service demand and not on the care itself. Now it makes sense to us, we can, with certainty, say in which health model we operate, or are seeking to operate. Now it is clear what is the direction (NGC^‡^4).
Strengthened constituent elements of NC*	All the tools we saw, the Systematization of Nursing Care, nursing diagnoses, the use of technologies, the tools that the information system offers that we did not use, we did not know the importance and how much it qualifies the NC* **[...]** *scientific knowledge regarding the definitions of nursing, person, environment, care, health; about the ICNP* ^*||*^ , which we did not know how to use, to prepare a diagnosis. Knowing and discussing all of this and the healthcare models was essential at this time of change **(NGC** ^‡^ **9).**
Concerns and desires for change in practice	*We saw ourselves in many examples cited and realized that we need to change! Deconstruct our own preconceptions to transform our practice. We do not feel ready yet, but we realize the need to consider what the person brings to set goals with them* **(NGC** ^‡^ **6).**
Resignified constituent elements of NC*	*Now everything makes sense! The consultation is with people who live with diabetes. We had to rethink this, about the context in which it is inserted and our professional role. We did not really consider the relationship with the user! But considering them as part of the diabetes care process is very important. This has been resignified. Because we thought we considered it, but in practice, our actions were prescriptive. We did not consider the user as an actor in the process, their real needs, their perspective as the agent of change. Our practice was like this and it was resignified. Teamwork. We talked a lot about this, “It is not healthy to centralize everything on ourselves, that is what causes overload”, and it was possible to resignify the importance of working as a team* **[...] (NGC** ^‡^ **9).**
Possibilities to minimize challenges	*Doing things without planning has been our practice, and we have realized that it is necessary to establish the division of tasks, plan in a short space of time, analyze the situation. And dividing and delegating tasks will make all the difference in achieving a very good result and not causing the work overload that we feel so much. Regarding communication, we concluded that what we want may be clear to us, but it may not be clear to the team and the user, and paying attention to this is essential* **(NGC** ^‡^ **5).**
Transformations in the practice of NC* with people with DM^†^	Our meetings have helped a lot! We have tried to put this into practice, and the consultation has improved a lot! Users feel valued by the changes we have introduced. The NC* has a different tone, it seems to have become more significant for them **[...].** *Revisiting our knowledge about nutrition was essential. This is something that we often do not even take into account, and here, another challenge, not only the nutrition of the person with DM* ^*†*^ , but healthy nutrition for the whole family! **(NGC** ^‡^ **7).** After we started delegating some tasks to the team, we realized that we were able to dedicate more time to the NC*. I feel that it has improved **(NGC** ^‡^ **9).**

*NC = Nursing consultation; ^†^DM = Diabetes *mellitus*; ^‡^NGC = Convergence group narrative; ^§^MACC = Chronic Conditions Care Model; ^||^ICNP = International classification for nursing practice

## Discussion

People experiencing a chronic condition such as diabetes require appropriate care from the onset of diagnosis, so they can undergo a favorable adaptation process to their new health condition and maintain the balance of their well-being^24^. Therefore, various organizational models are used with the aim of educating and supporting self-management for individuals with chronic diseases, with the Chronic Care Model (CCM) being the most widely used, as highlighted in a review study^25^. 

It is important to mention that the MACC has its foundations based on the CCM and, in addition to being an organizational model, it encourages changes in paradigms in care, aiming at comprehensive care, with the user at the center of care^10^. In this research, elements of CE with people with DM were highlighted that diverge from the assumptions of MACC, as a hegemonic view of the nurse-patient relationship is observed, with disease-centered and curative practices. Recent international research explored nurses’ perceptions of family-centered care for adults with diabetes and, similarly, the dominant medical model remains a challenge to overcome^26^.

In light of this reality, Brazilian nurses must reflect on the importance of their ethical and political role in caring for individuals with chronic diseases, in fulfilling and strengthening the principles of the *Sistema Único de Saúde* (SUS), as well as in enhancing the visibility of the profession. The way in which the participants of this study understand and operationalize the MACC differs exponentially from other realities, such as Austria, where nurses are recognized for their competence and are preferred by users to lead chronic care programs/models. This has practical implications for how care models are staffed and presents an opportunity for the nursing workforce to play a leadership role in service delivery^27^. 

In Singapore, patients receiving diabetes care in the primary health care system who adopt CCM also reported satisfaction with the nursing care provided, continuity of care, and the patient-centered approach involving goal setting and problem solving^28^.

However, the nurses in this study identified several challenges in carrying out NC with people who have DM related to the work process, such as overload, overlapping management and care duties, and the need to implement protocols. To overcome these challenges, Bolivian nurses identified the need for support in the care of people with diabetes at the primary level, encompassing four dimensions: organization of care and health policies; strengthening the knowledge, skills and attitudes of the health team; training of people living with diabetes and their families; and education about diabetes at the community level^29^.

The challenges highlighted in this research could negatively impact the quality of care provided and, consequently, lead to metabolic imbalance and complications such as diabetic foot and limb amputations in people with diabetes. Thus, effective multidisciplinary management of the clinical condition of diabetes is necessary, which requires adequate recruitment of health professionals, a well-defined team, adherence to evidence-based guidelines, a good infrastructure for data collection and mechanisms for quality improvement based on the review of clinical data^30^. 

In this research, the CCR method allowed nurses to reflect on their practice in NC with people who have DM and to initiate a process of change. In this way, the recognition by nurses of the need to overcome challenges occurred through an educational process that stimulated reflection and critical thinking, making them active agents and transformers of reality, which contributed to the establishment of strategies to minimize them. Similarly, studies carried out using the same method in other healthcare areas also enabled investments in in-service training, aiming to qualify nursing care^31-32^. 

The research participants redefined the practice of NC with people who have DM, understanding the nurse’s practice as the art of continuously and thoroughly perceiving the needs of the person being cared for, valuing the subjective aspects, which will allow them to reveal the person in their entirety, resulting in the choice of the best intervention for them^18^. 

The professional attitude was perceived as a need for change, being resignified as co-responsible care, which the participants called “caring with the patient”. Scientific evidence indicates that a centered professional-user relationship, with a dialogical approach, which prioritizes the emancipation of the subject and biopsychosocial aspects to determine care needs, enhances care for people with DM^33^ ^34^ ^35^. 

The perception of the need for professional training and qualification was fundamental. Continuing education in health is an integral part of the work process and allows the development of skills and abilities consistent with the care model recommended by the SUS^36^. Furthermore, when nurses receive structured training and support, and use treatment algorithms and technologies, nursing care becomes more effective, impacting glycated hemoglobin values, with reductions of 0.03 to 2%^37^. 

The demands for theoretical deepening of MACC and CE raised by the participants were conducted in a way that ensured significant learning based on reality^38^, resulting in satisfaction with the methodology used. This type of learning enables the improvement of critical thinking, autonomy, motivation to learn, active attitude, ability to work in a team and solve problems. To this end, we agree that nurses, during their ongoing training process, can develop skills to act effectively in PHC, reflecting on and about their practice and deciding on the need to remain in constant training to deal with issues arising from this practice^39^. 

It is worth highlighting the importance given by participants to discussions about scientific knowledge in nursing, resuming conceptual differences in the systematization of nursing care and the nursing process (NP), as well as the use of standardized language systems, that is, knowledge and tools that qualify NC with people who have DM. In 2024, the *Conselho Federal de Enfermagem* (COFEN) resolution nº 736, was published, which, in its Article 2, conceptualizes and qualifies the NP as a method that guides the nurse’s critical thinking and clinical judgment for care, which must be based on theoretical support, such as care models, standardized language systems, validated risk prediction assessment instruments and protocols^40^. Hence, it can be inferred that the aforementioned concept is strongly linked to what nurses experience during the educational-reflective process.

In this study, it was essential to obtain the result that the participants perceived that the NP aims to positively interfere in nursing care for people with DM, as it directs them towards clinical reasoning and the prescription of nursing care, aiming at a higher quality of care. As evidenced in a systematic review study, it is necessary to develop more research that analyzes the use of the nursing process in community health and public health contexts and, similarly, that evaluates nursing interventions and the population’s satisfaction with the NP^41^.

Another result points to the review of work management, with a desire expressed by participants to adopt a practice of interprofessional collaboration, when they cited some of its characteristics, such as teamwork, communication, division of tasks and planning. Norwegian PHC nurses recognized that learning interprofessional collaboration skills represents a significant opportunity for quality improvement in PHC services^42^.

In this systematic process of construction and reconstruction of praxis, developments occurred in the context of nurses, such as the construction of the guide for the development of CE with people with DM, which was later validated^43^, and the implementation of *Conselho Regional de Enfermagem do Rio Grande do Sul* (Coren/RS) nursing protocols through Municipal Decree nº 067/2023. In this way, participatory study designs are strongly supported, for the possibility of identifying innovative solutions with the potential to improve person-centered care for diabetes^44^. From this perspective, it is fundamentally important in the construction of educational technologies to encourage a high level of involvement of people with diabetes as an equally interested party^45^.

Therefore, this research has some strong points that are similar to a previous study that also used CCR^46^, such as the fact that it considers the reality and experience of participants in their workplace, which favors the content and its feasibility, expressing the potential for translating knowledge into practice. 

In this sense, the transformation of practice generated direct satisfaction for the user, and consequently professional appreciation. Therefore, it is confirmed that in the spaces where people meet, the hierarchical barriers implied by biomedical logic are broken, there is a democratization of health knowledge, a resignification throughout the entire trajectory and a constant movement of action-reflection-action^47^. We believe that the strategies used in this research that culminated in positive transformations in the work process of FHS nurses can be adapted and applied in other realities.

The limitations of this research are attributed to two factors. The research design did not include the participation of users, which could have triangulated the results brought by the nurses. The research conducted, even though it is a CCR, does not provide for the continuity of monitoring the NC to track the quality improvements achieved.

The choice of participatory analysis for the CCR represents an advancement in the scientific knowledge of nursing, as it enabled nurses to adopt the researcher’s perspective. Furthermore, allowing participants to discuss and review the collected data is a methodological improvement in qualitative research, and in this study, associated with CCR, it proved to be a feasible possibility. 

## Conclusion

The Nursing Consultation conducted in the FHS with people who have DM was carried out by nurses through elements that align with the MACC, such as establishing a bond with users, accountability, longitudinal care, and the social and family approach. However, they diverged from the assumptions of this model when there was a limited understanding of the care model, in addition to the professional attitude of blaming the user and the weaknesses in the application of the nursing process with people who have DM. These elements that portrayed the need for change in care practice were the starting point to support the educational process developed with the participants through the CCR.

Convergent Care Research has been developed over a long period of time and has allowed for the promotion of an action-reflection-action process with positive results for the translation of knowledge, such as: nurses who are aware and willing to change, interested in the theoretical and practical deepening of MACC and NP, and collaborative for the construction and implementation of instruments and technologies that favor the work process (protocol and guide). It can be seen, then, that the transformations that have occurred benefit users and contribute to the qualification of nursing care in the FHS. Therefore, research that demonstrates this commitment to articulating theory and practice is relevant in the field of Nursing, as it breaks away from the utilitarian idea of research and, in fact, promotes changes and brings innovations to the health sector.
