# Supplementary material for: Nursing consultation and diabetes: an educational and transformative process for primary health care
Source: Rev Lat Am Enfermagem. 2025 Feb 17;33:e4464. doi: 10.1590/1518-8345.7546.4464 (PMC11835004; doi:10.1590/1518-8345.7546.4464)
Supplement: Translation

# Translation: Consulta de enfermería y diabetes: proceso educativo y transformador para los cuidados primarios de salud^*^

**DOI:** 10.1590/1518-8345.7546.4463

**Authors:** Silvana de Oliveira Silva, Andréa Carvalho Araújo Moreira, Alexa Pupiara Flores Coelho Centenaro, Nara Marilene Oliveira Girardon-Perlini, Teresinha Heck Weiller, Maria Denise Schimith

**Affiliations:** 1 Prefeitura Municipal de Santiago, Secretaria Municipal de Saúde, Santiago, Rio Grande do Sul, RS, Brasil.; 2 Universidade Federal de Santa Maria, Santa Maria, Rio Grande do Sul, RS, Brasil.; 3 Universidade Estadual Vale do Acaraú, Sobral, Ceará, CE, Brasil.; 4 Universidade Federal de Santa Maria, Palmeira das Missões, Rio Grande do Sul, RS, Brasil.

**Keywords:** Proceso de Enfermería, Diabetes Mellitus, Atención Primaria de Salud, Modelos de Atención de Salud, Enfermedad Crónica, Aprendizaje Basado en Problemas.

## Abstract

**Objetivo:**

analizar los elementos constitutivos que sustentan la consulta de enfermería con personas que tienen diabetes *mellitus*, y desarrollar un proceso educativo basado en los supuestos del Modelo de Atención a las Condiciones Crónicas y mediado por un proceso reflexivo-dialógico con enfermeros(as), con el fin de mejorar la calidad de la práctica en la Estrategia de Salud de la Familia.

**Método:**

investigación convergente asistencial realizada con 12 enfermeros. Se utilizaron observación participante, entrevista individual semiestructurada y grupos de convergencia para la recolección de datos, los cuales fueron tratados mediante análisis participativo, con enfoque interpretativo.

**Resultados:**

la promoción del autocuidado, el establecimiento de vínculo profesional-usuario y el apoyo a cambios de hábitos de las personas con diabetes se acercaron al Modelo de Atención propuesto. Por otro lado, se identificaron como distantes las actitudes, valores y conocimientos que debilitan el cuidado. Los puntos de convergencia para mejorar la calidad de la consulta fueron la profundización teórica y práctica del modelo de atención, la diabetes, el enfrentamiento a la sobrecarga de trabajo, y la implementación de una guía y de un protocolo para el desarrollo de las consultas de enfermería. También se constató el fortalecimiento de la autonomía de los enfermeros, el despertar del pensamiento crítico, y la búsqueda de perfeccionamiento y resignificación de la relación con el usuario.

**Conclusión:**

la consulta de enfermería fue mejorada a partir de la participación activa de los enfermeros en un proceso educativo, reflexivo y dialógico.

## Destacados

**(1)** La Consulta de Enfermería para personas con DM por parte de enfermeras estuvo conformada por elementos cercanos y se distanciaron del MACC. **(2)** Elementos cercanos con MACC: el vínculo, la responsabilización, el cuidado longitudinal y el enfoque social y familiar. **(3)** Elementos se distanciaron del MACC: comprensión limitada del modelo, la postura profesional de culpabilización del usuario y de las debilidades del proceso de enfermería con las personas que tienen DM. **(4)** La consulta de enfermería fue mejorada a partir de la participación activa de los enfermeros en un proceso educativo, reflexivo y dialógico.

## Introducción

La diabetes *mellitus* (DM) es una enfermedad crónica no transmisible que causó aproximadamente 2,0 millones de muertes en todo el mundo en 2019. En el período de 2000 a 2019, la DM registró un aumento global del 3% en el número de muertes y, en las Américas, superó el accidente cerebrovascular en la medida de años de vida ajustados por discapacidad, ocupando así el segundo lugar^1^.

Según la Federación Internacional de Diabetes (FID), la prevalencia de la enfermedad en los países de ingreso bajo es del 5,5%^2^. En Brasil, se identificó una incidencia del 8,6%, siendo el 68,2% de la población consciente de su diagnóstico, el 92,2% de los que conocen su diagnóstico siguen tratamiento farmacológico, y de estos, solamente el 35,8% tiene niveles controlados de hemoglobina glicosilada^3^. 

Por lo tanto, es fundamental prestar atención a los daños actuales y futuros de esta enfermedad, para planificar la implementación de políticas públicas efectivas, ya que la mayoría de los países han avanzado poco en el cumplimiento de la meta de Desarrollo Sostenible 3.4, que prevé una reducción de un tercio de la mortalidad prematura por enfermedades no transmisibles entre 2015 y 2030. En este contexto, el rastreo y el tratamiento de la DM son intervenciones de alta prioridad para reducir la mortalidad por enfermedades crónicas^4^. 

La Atención Primaria de Salud (APS) en Brasil, organizada a nivel nacional por medio de la Estrategia de Salud de la Familia (ESF), debe ser la puerta de entrada preferencial para las personas con DM en el sistema de salud. Es el punto de atención estratégico para satisfacer mejor las necesidades de salud, incluido el seguimiento longitudinal y continuo^5^. Los tratamientos para la DM se basan en enfoques no farmacológicos y farmacológicos para el control glucémico, que deben ser elegidos considerando los aspectos biopsicosocioespirituales de cada persona, por medio de una relación interpersonal entre el profesional y el paciente, ya que esto influye directamente en la adherencia al tratamiento.

Por tanto, el enfermero es el profesional de primer contacto con el paciente, quien debe asumir un papel de liderazgo en la detección, tratamiento y rehabilitación de la diabetes. Este profesional también es responsable de apoyar los esfuerzos para prevenir la enfermedad y promover la salud de las personas que viven con diabetes, permitiéndoles así participar en la toma de decisiones relacionadas con su tratamiento^6^. 

Para la Organización Mundial de la Salud (OMS), la contribución de la enfermería para prevenir y gestionar la diabetes en el sistema de salud se demuestra por medio de seis características: 1) coordinación de los cuidados para garantizar que las necesidades de salud del paciente se satisfagan a lo largo del tiempo; 2) participación en un enfoque multidisciplinario de cuidados basado en una relación integrada entre los profesionales de la salud, permitiendo a diferentes profesionales trabajar en equipo para mejorar la calidad de los cuidados; 3) movilización y capacitación del personal de enfermería para especializarse en enfermedades no transmisibles, con el objetivo de lograr tratamientos más eficaces y sostenibles; 4) mejor acceso a los cuidados; 5) empoderamiento de los individuos y de la comunidad; y 6) uso de la tecnología para mantener el acceso a los servicios de salud esenciales y reducir la exposición a la COVID-19^7^.

Hay esfuerzos de enfermeros brasileños para contribuir a la adherencia al tratamiento de la DM, proporcionando bases científicas para los cuidados de enfermería, con el fin de orientar acciones que promuevan una mayor adherencia al tratamiento con la participación activa del paciente, reduciendo las complicaciones, las hospitalizaciones y la mortalidad^8^. Por otro lado, un estudio internacional^9^ demostró que la diabetes aún no es una prioridad en los centros de atención primaria. Los enfermeros explicaron la importancia de trabajar en la prevención primaria de la diabetes, priorizando el cuidado de las personas con prediabetes, y señalaron la necesidad de establecer directrices clínicas, apoyo en conocimientos y habilidades asistenciales, además de la implementación de un proceso de trabajo interprofesional, aspectos cruciales en la atención de la salud de las personas con diabetes, que se basa en el Modelo de Atención a las Condiciones Crónicas (MACC).

El MACC fue propuesto por Eugênio Vilaça Mendes para atender las especificidades del sistema de salud pública brasileño. Tiene su origen en el Modelo de Cuidados Crónicos (MCC) proveniente de los Estados Unidos, e incorpora otros dos modelos: el Modelo Pirámide de Riesgo (MPR) y el Modelo de la Determinación Social de la Salud, para adaptarse a las exigencias de un sistema de atención a la salud público y universal como el *Sistema Único de Saúde* (SUS)^10^. Es un instrumento operativo, establecido en el Plan Nacional de Enfrentamiento de Condiciones Crónicas para alcanzar los objetivos de desarrollo del milenio al 2030.

La consulta de enfermería (CE) orienta el pensamiento crítico y el juicio clínico del enfermero, orientándolo al cuidado de la persona, familia, comunidad y grupos especiales. En el contexto de la ESF y del cuidado a las personas con enfermedades crónicas, la CE favorece una atención resolutiva, proactiva y humanizada, basada en acciones educativas capaces de empoderar a los usuarios para alcanzar los objetivos establecidos en el proceso de cuidar y autocuidado.

Sin embargo, son preocupantes las dificultades encontradas en la práctica de la CE en la APS, tales como: falta de tiempo, falta de agilidad para el diagnóstico de enfermería, problema en la interfaz entre asistencia y gestión, y excesivas exigencias burocráticas^11^.

En el campo científico, hay mucho por investigar sobre la consulta de enfermería con personas con diabetes en el contexto de la APS, dada su relevancia, especialmente cuando está asociada a los supuestos del MACC. Son pocos los estudios sobre este tema, que apuesta por promover cambios y/o innovaciones en la práctica del cuidado, adoptando una perspectiva constante de construcción del pensar y del hacer.

Teniendo en cuenta lo anterior, esta investigación tiene como objetivo analizar los elementos constitutivos (conocimientos, tecnologías, creencias y valores) que sustentan la consulta de enfermería con personas que tienen diabetes *mellitus*, y desarrollar un proceso educativo basado en los supuestos del Modelo de Atención a las Condiciones Crónicas y mediado por un proceso reflexivo-dialógico con enfermeros(as), con el fin de mejorar la calidad de la práctica en la Estrategia de Salud de la Familia.

## Método

### Diseño del estudio

Investigación convergente asistencial (ICA) que tiene como objetivo, durante el transcurso de la investigación, promover cambios y/o innovaciones en la práctica del cuidado, a partir de la construcción continua del pensar y del hacer^12^. Su elaboración cumplió con las recomendaciones de los Criterios Consolidados para Informes de Investigación Cualitativa^13^. 

### Lugar del estudio

El estudio se realizó en una ciudad de tamaño mediano ubicada en la región centro-oeste del estado de Rio Grande do Sul, Brasil, que forma parte del proyecto de Planificación de la Atención en Salud y cuenta con 11 unidades de ESF, que cubren el 86% de la población total.

### Período

La producción de los datos se realizó entre agosto de 2020 y diciembre de 2021, período durante el cual la pandemia de COVID-19 azotaba al país. Se tomaron todas las medidas de prevención de transmisión. 

### Participantes y criterios de selección

Se incluyeron enfermeros(as) que trabajaban en ESF y con experiencia en CE con personas con DM de al menos 6 meses [según el artículo 442-A de la Consolidación de las Leyes del Trabajo (CLT) de Brasil, este es el tiempo máximo de experiencia que puede ser requerido por los empleadores para fines de contratación]. Se estableció como criterio de exclusión: enfermeros(as) que por algún motivo estuvieran alejados(as) del trabajo por más de 30 días durante el período de recolección de datos, lo cual no ocurrió. De este modo, 12 enfermeros(as) fueron contactados por correo electrónico para una reunión de presentación de la propuesta y, en su totalidad, aceptaron participar en el estudio. 

### Recolección de datos

Se adoptó la triangulación de técnicas para la producción de datos: observación participante activa^14^, entrevista semiestructurada^15^ y grupos de convergencia^16^. 

El objetivo de la observación participante fue captar y registrar los más variados elementos e impresiones sobre la realización de la CE con personas con DM. Así, se utilizó un guión preestablecido que incluyó los siguientes aspectos: relación profesional con el usuario, comunicación, etapas del proceso de enfermería, registro de enfermería, y ambiente.

La interacción de la investigadora durante las observaciones se produjo de forma gradual. Durante la CE, buscó mantener una posición más pasiva, interactuando estrictamente lo necesario. La participación activa ocurrió mayoritariamente después de finalizar la consulta, cuando, entre una cita y otra, se dialogaba sobre el caso y se compartían conocimientos a partir de las percepciones de la investigadora y de las dudas planteadas por los participantes.

La interrupción de las observaciones se estableció mediante el criterio de saturación teórica^17^, que consistió en una recopilación individual, buscando identificar si los elementos propuestos por el guión fueron explorados. Una vez que todos los elementos fueron contemplados y no emergió ninguna otra información nueva pertinente al tema del estudio, se cerraron las observaciones. Con un total de 120 horas, y con un promedio de 10 horas/participante, los datos producidos en esta etapa fueron registrados en un diario de campo y en una grabadora digital, inmediatamente después de su finalización, codificados y posteriormente transcritos al editor de textos *Microsoft Word*. 

La entrevista individual semiestructurada fue realizada por la investigadora después de alcanzar la saturación de las observaciones y antes de los grupos de convergencia. Para ello, se les animó a hablar del día a día de la CE con personas con DM, abordando aspectos como objetivo, qué hace y cómo lo hace; sobre el MACC, su conocimiento y aplicabilidad en la vida cotidiana; sobre los desafíos y las potencialidades de la realización de la CE con personas con DM; y sugerencias para los grupos de convergencia. Se realizó una prueba piloto, pero no formó parte de la muestra de participantes. Se realizaron 12 entrevistas, previamente programadas con los(as) participantes, en las unidades de salud, audiograbadas, con una duración promedio de 40 minutos. Luego, un asistente de investigación capacitado transcribió el material al editor de texto de *Microsoft Word*.

Los datos obtenidos por medio de estas dos técnicas sustentaron el Grupo de Convergencia (GC), que tiene como finalidad realizar la investigación simultáneamente con la práctica asistencial junto al colectivo, con enfoque en la educación para la salud o en la práctica clínica. En este espacio, se busca promover discusiones sobre un determinado tema, foco de la investigación, en un proceso interactivo entre participantes e investigador, de manera que se permita la dialogicidad^18^. 

Los encuentros se desarrollaron según cronograma elaborado con los(as) participantes, en la sala de reuniones de la Secretaría Municipal de Salud, con un tiempo promedio de 2 horas y 30 minutos. Fueron grabados en audio en una grabadora digital y la investigadora contó con el apoyo de un asistente de investigación, estudiante de la Maestría en Enfermería, quien recibió capacitación y contribuyó activamente en la planificación, ejecución, observación y transcripción. 

Para lograr la cohesión, el grupo de convergencia pasó por un proceso de cuatro fases, denominado Proceso de los Cuatro Erres (R): reconocimiento; revelación, repartir y repensar^16^. La fase reconocimiento tuvo como finalidad posibilitar el primer encuentro del grupo y establecer relaciones entre los participantes y la investigadora para que la cohesión del grupo pudiera darse por medio del diálogo participativo. Esta fase abarcó dos encuentros. El primero tuvo como objetivo analizar el conocimiento de los(as) participantes sobre la CE en términos de su finalidad y supuestos. Para ello se realizaron actividades grupales, incluyendo la creación de carteles, dramatizaciones y debates. El segundo encuentro se centró en el conocimiento sobre el MACC, utilizando palabras clave e imágenes como recursos para promover la interacción y el diálogo.

La fase revelación comprendió el momento en que los participantes se identificaron con sus pares por la experiencia compartida con la propuesta de los encuentros. Este proceso se desencadenó cuando se les animó a hablar sobre sus vivencias con la CE con personas con DM y elaborar los conceptos que rigen su práctica asistencial. 

La fase repartir fue el momento en el que el grupo, mediado por el intercambio de experiencias, fue llevado a tomar decisiones compartidas sobre el propósito de la reunión. Esta fase contó con tres encuentros y con una participación más activa de la investigadora. Las actividades se desarrollaron con apoyo de lectura, discusión de materiales científicos, dramatizaciones, estudio de casos y apoyo matricial por parte de un nutricionista.

La fase repensar se caracterizó por la reflexión del grupo sobre las implicaciones de los problemas identificados y la posibilidad de transferir lo compartido y aprendido para el cotidiano de los(as) participantes^16^. Esta fase se desarrolló en tres momentos, utilizándose estrategias como: técnica de *Brainstorm*, estudio de casos, dinámicas de grupo y elaboración de la guía para la CE con personas que tienen DM.

Los encuentros grupales fueron planificados y desarrollados tomando como referencia la Pedagogía Liberadora de Paulo Freire, la cual ha sido utilizada en estudios en las áreas de educación, salud y enfermería. En este punto, la Investigación Convergente Asistencial se articula con este marco, ya que se fundamenta en el diálogo crítico liberador, basado en el principio de que la reflexión crítica conduce a la acción (práctica), llamada praxis^19^. 

### Análisis de los datos

El análisis se fundamentó en una investigación participativa con enfoque interpretativo, realizada por medio de la construcción y retroalimentación de narrativas^20^. Basado en los presupuestos de la Teoría Hermenéutica de Gadamer, este enfoque considera que los actos de interpretación son dialógicos, explorando estrategias participativas y sugiriendo algunos cambios en la forma clásica de llevar a cabo las investigaciones. Entre esos cambios, se utilizan dos estrategias: la construcción de narrativa y validación, y la producción de consenso participativo^(20)^. De esta manera, el participante actúa en el proceso de investigación, lo que permite la resignificación y la autorreflexión de sus conductas^21^. 

A lo largo del proceso de transcripción y organización de los datos, se realizaron diversas lecturas en profundidad con el fin de comprender y explorar nuevos testimonios. Así, fue posible percibir relatos reincidentes, peculiares, divergentes y contradicciones expresadas que involucraban la práctica de la CE, y hacer las primeras inferencias e interrogantes. 

Teniendo en cuenta estas consideraciones, el análisis se realizó de la siguiente manera: luego de transcribir cada una de las entrevistas semiestructuradas (n=12), se construyó una narrativa. A continuación, la investigadora regresó al campo y leyó la narrativa a cada participante y, por medio del proceso dialógico, el(la) participante reflexionó sobre la narrativa y tuvo la oportunidad de cuestionarla o reforzarla y validarla. Con los grupos de convergencia, de igual manera, se produjeron nueve narrativas, una de cada encuentro, las cuales fueron leídas y validadas con los participantes a medida que se desarrollaban los encuentros. Siempre antes de iniciar el próximo grupo, se reforzaba la posibilidad de coproducción de los(as) participantes.

Como forma de facilitar la organización de esos datos y la categorización de los núcleos argumentales, las notas de observación y las narrativas de las entrevistas y de los grupos fueron insertadas en el *NVivo Software* Corporativo. Así, con el desarrollo de 21 narrativas y las notas de observación, fue posible construir y encadenar los núcleos argumentales, los cuales buscaban responder a la pregunta de investigación^20^.

### Aspectos éticos

La investigación siguió los lineamientos de la Resolución nº 466/12 del Consejo Nacional de Salud^22^, además de la Resolución nº 510/2016 del Ministerio de Salud, que enfatiza las especificidades éticas de la investigación en ciencias humanas y sociales y de otras que utilizan metodologías propias de estas áreas^23^. Fue aprobada por el Comité de Ética en Investigación de la *Universidade Federal de Santa Maria*, bajo dictamen nº 4.209.003. Todos(as) los(as) participantes aceptaron participar en el estudio y firmaron el Formulario de Consentimiento Informado. El anonimato está garantizado con los siguientes códigos: O (Observación) seguido de la letra E (enfermero), numeral del 1 al 12 y fecha (día/mes/año); NE (Narrativa Enfermero) seguido del numeral del 1 al 12; NGC (Narrativa Grupo de Convergencia) seguido del numeral del 1 al 9. 

## Resultados

Participaron del estudio 12 enfermeras, la mayoría mujeres (n=11), con una edad promedio de 42 años (mínima de 33 años y máxima de 51 años). El tiempo promedio de formación en Enfermería fue de 12 años (mínimo de 5 años y máximo de 25 años), y el tiempo de trabajo en la ESF fue, en promedio, de 7,5 años, siendo el menor tiempo de 6 meses (n =1) y el mayor de 11 años (n=3).

Los resultados se presentarán por medio de los siguientes núcleos argumentales: Elementos constitutivos de la Consulta de Enfermería con personas que tienen DM que se acercan y se distancian de los presupuestos del Modelo de Atención a las Condiciones Crónicas; y Ahora tiene sentido: la convergencia entre la investigación y el proceso educativo.

### Elementos constitutivos de la Consulta de Enfermería con personas que tienen DM que se acercan y se distancian de los presupuestos del MACC

Los elementos de la CE con personas que tienen diabetes en la ESF abarcan características que se acercan y se distancian del MACC, como se demuestra en la Figura 1.

**Figura 1:** Elementos constitutivos de la Consulta de Enfermería con personas que tienen diabetes *mellitus*

*CE = Consulta de enfermería; ^†^DM = Diabetes *mellitus*; ^‡^MACC = Modelo de Atención a las Condiciones Crónicas

Los(as) enfermeros(as) enfrentan algunos desafíos en la realización de la CE con personas con diabetes que han durado algunos años, a saber: sobrecarga de trabajo, falta de protocolos de enfermería y procedimientos operativos estándar (POEs), y cantidad insuficiente de profesionales de enfermería para satisfacer las demandas de atención y gestión. [...] *¡Creo que lo que más limita la asistencia al usuario durante las consultas es la sobrecarga! Estoy aquí para ayudar al usuario, ¡pero eso no es todo lo que hago! Hay problemas de gestión del servicio, demanda espontánea y administrativa* [...] (NE3). [...] *En varias ocasiones, la enfermera fue interrumpida durante la consulta para resolver cuestiones administrativas del servicio, como solicitar materiales, recibir insumos, hacer contacto con otro servicio, informar sobre el período de vacaciones del agente comunitario; pidió permiso y salió de la sala para evaluar una lesión en la sala de curaciones* (NO 08/09/2020)*. Reconocemos la necesidad de desarrollar e implementar los POEs y protocolos de enfermería, porque cada día asistimos a situaciones en las que, si tuviéramos estos instrumentos, tendríamos más autonomía* [...] *sin mencionar mayor calidad en la atención al paciente. De esta manera nuestro tiempo sería mejor aprovechado* [...]*. Por estos motivos, mirando el tema de la resolución en atención primaria, entendemos que una de las posibilidades son los protocolos, los POEs.* (NGC1). *Incluso antes de la pandemia, el número de profesionales de enfermería ya era insuficiente. Ya no podíamos manejarlo porque en realidad necesitaríamos una enfermera para la parte asistencial y otra para la gestión. Y solo tenemos dos técnicos de enfermería en el equipo, sin embargo, a menudo hay uno que no está presente, porque o bien está de vacaciones, con un informe, con una licencia, de descanso, o en otra actividad, como ahora, que está asignado a otra unidad para apoyar en la vacunación.* (NE10).

Por otro lado, los(as) participantes reconocieron algunos factores contextuales como medios para potenciar la consulta de enfermería en la ESF para personas con diabetes, como el apoyo de la gestión municipal, red municipal de atención de salud, y la compartición del cuidado con el ambulatorio de especialidades; el reconocimiento de la población al desempeño profesional de los enfermeros; y el vínculo con los usuarios y la relación profesional con el médico de la unidad: [...] *me siento contemplada en esta gestión. Nuestras demandas son atendidas; tenemos horario protegido para reuniones de equipo y capacitaciones.* (NE9). *Lo que potencia la consulta es que la atención primaria está muy fortalecida en la ciudad.* [...] *Los usuarios tienen acceso, contención, reconocen la unidad como puerta de entrada y nuestro trabajo como enfermeras* [...] (NE1). *La cuestión de las interconsultas con la médica, ya que ayuda en la resolutividad* (NE4). [...] *contamos con un ambulatorio especializado para derivar a los usuarios que necesitan evaluación* [...] *y siempre nos retroalimentan para implementar el cuidado* (NGC3). [...] *también disponemos de una red de atención a la salud para los usuarios aquí en la ciudad, como laboratorios, centro de referencia de asistencia social, el núcleo de apoyo a la salud familiar, nutrición, y todo esto podemos poner a su disposición en la Consulta, el usuario tiene acceso siempre que lo necesita* (NE7).

### Ahora tiene sentido: la convergencia entre la investigación y el proceso educativo 

De la convergencia entre la investigación y la práctica asistencial emergieron las demandas para cualificar la Consulta de Enfermería en la ESF con personas que tienen DM. El proceso educativo desarrollado a lo largo de los grupos de convergencia está representado en la Figura 2.

**Figura 2:** Puntos de convergencia entre investigación y práctica asistencial

*CE = Consulta de enfermería; ^†^MACC = Modelo de Atención a las Condiciones Crónicas; ^‡^DM = Diabetes *mellitus*

En este estudio, el intercambio de conocimientos entre investigadora y participantes subsidia acciones concretas de cambio y movimientos de reflexión-acción que resultaron en la construcción colectiva de una guía para la CE con personas que tienen DM y la implementación de un protocolo de enfermería. Tales cambios están representados en la Figura 3.

**Figura 3:** Transformaciones en el cotidiano de la Consulta de Enfermería a partir del proceso educativo-dialógico

Percepción de la necesidad de profundización teórica y práctica de la CE*	[...] *Necesitamos profundizar nuestro conocimiento sobre DM* ^*†*^ y cuidado con los usuarios. Instituir un protocolo de enfermería y una guía de CE* para ayudarnos en nuestro día a día y estructurar mejor la CE*, todo el proceso, recolección de datos e intervenciones en marcos teóricos (NGC^‡^7). [...] *En los talleres de planificación, la forma de presentar el MACC* ^*§*^ fue más densa, el foco estaba en organizar la demanda del servicio y no en el cuidado mismo. Ahora tiene sentido para nosotros, podemos decir con certeza en qué modelo de atención sanitaria operamos o pretendemos operar. Ahora está claro cuál es el norte (NGC^‡^4).
Elementos constitutivos de la CE* fortalecidos	Todas las herramientas que vimos, la Sistematización de la Atención de Enfermería, los diagnósticos de enfermería, el uso de las tecnologías, las mismas herramientas que ofrece el sistema de información que no utilizábamos, no sabíamos la importancia y cuánto cualifica la CE* **[...] el** *conocimiento científico en relación a las definiciones de enfermería, persona, ambiente, cuidado, salud; sobre la CIPE* ^*||*^ que no sabíamos cómo utilizar, elaborar un diagnóstico. Conocer y discutir todo esto y los modelos de atención en salud fue fundamental en esta época de cambios **(NGC** ^‡^ **9).**
Inquietudes y deseos para el cambio de la práctica	¡Nos vimos en muchos ejemplos citados y nos dimos cuenta de que necesitamos cambiar! Deconstruir nuestras propias ideas preconcebidas para transformar nuestra práctica. Todavía no nos sentimos preparadas, pero nos damos cuenta de la necesidad de considerar lo que aporta la persona para fijar metas con ella (NGC^‡^6).
Elementos constitutivos de la CE* resignificados	¡Ahora todo tiene sentido! La consulta es con personas que viven con diabetes. Tuvimos que repensar esto, en cuanto al contexto en el que está inserto y nuestro rol profesional. ¡La relación con el usuario que no considerábamos mucho! Pero considerarlo como parte del proceso de atención de la diabetes es muy importante. Esto ha sido resignificado. Porque pensábamos que lo considerábamos, pero en la práctica, nuestras acciones eran prescriptivas. No considerábamos al usuario como actor del proceso, sus necesidades reales, su perspectiva como agente de cambio. Nuestra práctica era así y fue resignificada. Trabajo en equipo. Hablamos mucho de esto, “No es sano centrarnos todo en nosotros mismos, esto genera sobrecarga”, y se logró resignificar la importancia de trabajar en equipo [...] (NGC^‡^9).
Posibilidades de minimizar los desafíos	*Prescindir de la planificación ha sido nuestra práctica y nos damos cuenta de que es necesario instituir la división de tareas, planificar en un corto espacio de tiempo, analizar la situación. Y dividir y delegar tareas marcará la diferencia para lograr un muy buen resultado y no provocar la sobrecarga de trabajo que tanto sentimos. En cuanto a la comunicación, concluimos que para nosotros puede estar claro lo que queremos, pero para el equipo y el usuario quizás no, y prestar atención a esto es fundamental* **(NGC** ^‡^ **5).**
Transformaciones en la práctica de la CE* con las personas que tienen DM^†^	¡Nuestras reuniones han ayudado mucho! ¡Hemos intentado ponerlo en práctica y la consulta ha mejorado mucho! Los usuarios se sienten valorados con los cambios que hemos introducido. La CE* tiene un contenido diferente, parece haberse vuelto más significativa para ellos [...]*. Revisar los conocimientos sobre alimentación fue fundamental. Un cuidado que muchas veces ni siquiera nosotros tenemos, y aquí, otro desafío, no solamente la alimentación de la persona con DM* ^*†*^ , ¡sino una alimentación saludable para toda la familia! (NGC^‡^7). Después de que comenzamos a delegar algunas funciones al equipo, nos dimos cuenta de que era posible dedicarnos más a la CE*. Siento que ha mejorado (NGC^‡^9).

*CE = Consulta de enfermería; ^†^DM = Diabetes *mellitus*; ^‡^NGC = Narrativa grupo de convergencia; ^§^MACC = Modelo de Atención a las Condiciones Crónicas; ^||^CIPE = Clasificación internacional para la práctica de enfermería

## Discusión

Las personas que experimentan una condición crónica como la diabetes requieren de cuidados adecuados desde el inicio del diagnóstico, para que vivan un proceso de adaptación favorable a su nueva condición de salud y mantengan un bienestar equilibrado^24^. Por ello, se utilizan varios modelos organizativos con el objetivo de educar y apoyar el autocuidado de las personas con enfermedades crónicas, siendo el Modelo de Cuidados Crónicos (MCC) el más utilizado, como destaca un estudio de revisión^25^. 

Es importante mencionar que el MACC tiene sus fundamentos basados en el MCC y, además de ser un modelo organizacional, propicia cambios de paradigma en la atención, con miras a una atención integral, siendo el usuario el centro del cuidado^10^. En esta investigación, se destacaron elementos de la CE con personas con DM que se distancian de los supuestos del MACC, ya que se observa una visión hegemónica de la relación enfermero-paciente, con prácticas centradas en la enfermedad y curativistas. Una investigación internacional realizada recientemente exploró las percepciones de enfermeros sobre el cuidado centrado en la familia de personas adultas con diabetes y, de manera similar, el modelo médico dominante sigue siendo un desafío por superar^26^.

Frente a esta realidad, los enfermeros brasileños deben reflexionar sobre la importancia de su papel ético y político en el cuidado de las personas con enfermedades crónicas, en el cumplimiento y fortalecimiento de los principios del *Sistema Único de Saúde* (SUS), así como en la visibilización de la profesión. La forma en que los(as) participantes en este estudio entienden y operacionalizan el MACC difiere exponencialmente de otras realidades, como Austria, donde los enfermeros son reconocidos por su competencia y tienen preferencia de los usuarios para liderar programas/modelos de cuidados crónicos. Esto tiene implicaciones prácticas en la forma en que los modelos de cuidado se dotan de profesionales, y presenta una oportunidad para que la fuerza laboral de enfermería desempeñe un papel de liderazgo en la prestación de servicios^27^. 

En Singapur, los usuarios en seguimiento para diabetes en el sistema de atención primaria de salud que adoptan el MCC también informaron satisfacción con los cuidados de enfermería prestados, con la continuidad de los cuidados, y con el enfoque centrado en el paciente, que involucra el establecimiento de metas y la resolución de problemas^28^.

Sin embargo, los(as) enfermeros(as) en este estudio identificaron varios desafíos para realizar la CE con personas que tienen DM, relacionados con el proceso de trabajo, como sobrecarga, atribuciones gerenciales y asistenciales superpuestas, y la necesidad de implementar protocolos. Para superar estos desafíos, enfermeros bolivianos resaltaron la necesidad de apoyo en el cuidado de las personas con diabetes en el nivel primario, abarcando cuatro dimensiones: organización de los cuidados y políticas de salud; fortalecimiento de los conocimientos, habilidades y actitudes del equipo de salud; capacitación de las personas que viven con diabetes y sus familias; y educación sobre diabetes a nivel comunitario^29^.

Los desafíos resaltados en esta investigación podrían interferir negativamente con la calidad de la atención brindada y, en consecuencia, provocar una falta de control metabólico y complicaciones como el pie diabético y la amputación de extremidades en personas con diabetes. Así, es necesario un manejo multidisciplinario eficaz de la condición clínica de la diabetes, lo que requiere la contratación adecuada de profesionales de salud, un equipo bien definido, adhesión a directrices basadas en evidencias, una buena infraestructura para la recolección de datos y mecanismos para la mejora de la calidad basados en la revisión de datos clínicos^30^. 

En esta investigación, el método ICA permitió a los(as) enfermeros(as) reflexionar sobre su práctica en la CE con personas que tienen DM e iniciar un proceso de cambio. De esta manera, el reconocimiento por parte de los(as) enfermeros(as) de la necesidad de superar desafíos se dio a través de un proceso educativo que estimuló la reflexión y la crítica, convirtiéndolos en agentes activos y transformadores de la realidad, lo que contribuyó al establecimiento de estrategias para minimizarlos. De manera similar, estudios realizados con el mismo método en otras áreas asistenciales también permitieron invertir en la formación en servicio, con el objetivo de cualificar el cuidado de enfermería^31-32^. 

Los(as) participantes de la investigación resignificaron la práctica de la CE con personas que tienen DM, comprendiendo el ejercicio del(a) enfermero(a) como el arte de percibir de manera continua y minuciosa las necesidades del ser cuidado, valorando los aspectos subjetivos, lo que les permitirá desvelar a la persona en su integralidad, resultando en la elección de la mejor intervención para ella^18^. 

La actitud profesional fue percibida como una necesidad de cambio, siendo resignificada para un cuidado corresponsable, que los(as) participantes denominaron “cuidar con el paciente”. La evidencia científica indica que la relación profesional-usuario centrada, con una postura dialógica, que busca la emancipación del sujeto y los aspectos biopsicosociales para determinar las necesidades de cuidado, potencia la atención a las personas con DM^33-34-35^. 

La percepción de la necesidad de formación y cualificación profesional fue fundamental. La educación continua en salud es parte integral del proceso de trabajo y permite el desarrollo de habilidades y capacidades consistentes con el modelo de atención recomendado por el SUS^36^. Además, cuando los enfermeros reciben capacitación y apoyo estructurado, y utilizan algoritmos de tratamiento y tecnologías, el cuidado de enfermería se vuelve más efectivo, impactando los valores de hemoglobina glicosilada, con reducciones del 0,03 al 2%^37^. 

Las demandas de profundización teórica del MACC y la CE planteadas por los participantes fueron realizadas con el fin de garantizar un aprendizaje significativo basado en la realidad^38^, generando satisfacción con la metodología utilizada. Este tipo de aprendizaje permite mejorar el pensamiento crítico, la autonomía, la motivación para aprender, la actitud activa, la capacidad de trabajar en equipo y de resolver problemas. En este sentido, coincidimos con la prerrogativa de que los enfermeros, durante su proceso de formación permanente, puedan desarrollar habilidades para actuar eficazmente en la APS, reflexionando en y sobre la práctica y decidiendo sobre la necesidad de permanecer en constante formación para afrontar las problemáticas que surgen de esta práctica^39^. 

Vale la pena destacar la importancia que los(as) participantes dieron a las discusiones sobre el saber científico de la enfermería, retomando las diferencias conceptuales de sistematización de la asistencia de enfermería y el proceso de enfermería (PE), así como la utilización de sistemas de lenguajes estandarizados, es decir, saberes y herramientas que cualifican la CE con personas que tienen DM. En el año 2024 se publicó la resolución del *Conselho Federal de Enfermagem* (COFEN) nº 736, que, en su artículo 2^o^, conceptualiza y califica el PE como un método que orienta el pensamiento crítico y el juicio clínico del enfermero para el cuidado, el cual debe ser basado en soporte teórico, como modelos de cuidado, sistemas de lenguajes estandarizados, instrumentos de evaluación de predicción de riesgos validados y protocolos^40^. De esta manera, se puede inferir que el concepto antes mencionado está fuertemente vinculado al vivido por los enfermeros durante el proceso educativo reflexivo. 

En este estudio, fue fundamental obtener el resultado de que los(as) participantes percibieron que el PE tiene como objetivo interferir positivamente en la asistencia de enfermería a personas que tienen DM, ya que lo dirige al razonamiento clínico y a la prescripción de cuidados de enfermería, buscando una mayor calidad en la atención. Como se evidencia en un estudio de revisión sistemática, es necesario desarrollar más investigaciones que analicen la utilización del proceso de enfermería en contextos de salud comunitaria y salud pública y, de la misma manera, que evalúen las intervenciones de enfermería y la satisfacción de la población con el PE^41^.

Otro resultado señala la revisión de la gestión del trabajo, con un deseo manifestado por los(as) participantes de adoptar una práctica de colaboración interprofesional, al mencionar algunas de sus características, como el trabajo en equipo, la comunicación, la división de tareas y la planificación. Enfermeros de la APS de Noruega reconocieron que aprender las habilidades de colaboración interprofesional representa una oportunidad significativa para mejorar la calidad en los servicios de APS^42^.

En este proceso sistemático de construcción y reconstrucción de la praxis, ocurrieron desdoblamientos en el contexto de los(as) enfermeros(as), como la elaboración de una guía para el desarrollo de la CE con personas con DM, la cual fue validada posteriormente^43^, y la implementación de los protocolos de enfermería del *Conselho Regional de Enfermagem do Rio Grande do Sul* (Coren/RS), por medio del Decreto Municipal nº 067/2023. De esta manera, se apoya vigorosamente el diseño de estudios participativos, por la posibilidad de identificar soluciones innovadoras con potencial para mejorar el tratamiento centrado en la persona con diabetes^44^. Desde esta perspectiva, es de fundamental importancia en la construcción de tecnologías educativas favorecer un alto nivel de implicación de las personas con diabetes como parte igualmente interesada^45^.

Por lo tanto, se identifican en esta investigación algunos puntos fuertes que se asemejan a un estudio anterior que también utilizó la ICA^46^, como el hecho de considerar la realidad y la experiencia de los participantes en su lugar de trabajo, lo que favorece el contenido y su viabilidad, expresando el potencial de la translación del conocimiento para la práctica. 

En este sentido, la transformación de la práctica generó satisfacción directa para el usuario y, en consecuencia, una valorización profesional. Por lo tanto, se reafirma que en los espacios de encuentro entre las personas, las barreras jerárquicas implicadas en la lógica biomédica se rompen, hay una democratización del saber en salud, una resignificación a lo largo de toda la trayectoria y un constante movimiento de acción-reflexión-acción^47^. Se cree que las estrategias utilizadas en esta investigación, que culminaron en transformaciones positivas en el proceso de trabajo de los(as) enfermeros(as) de la ESF, pueden ser adaptadas y aplicadas en otras realidades.

Las limitaciones de esta investigación se atribuyen a dos factores. La participación de usuarios(as) no fue prevista en el diseño de la investigación, lo que podría haber triangulado los resultados aportados por los(as) enfermeros(as). La investigación desarrollada, aunque se trate de una ICA, no prevé un seguimiento continuo por parte de la CE para controlar la mejora de la calidad alcanzada.

La elección del análisis participativo para la ICA es un avance del conocimiento científico de la enfermería, ya que permitió que los(as) enfermeros(as) utilizaran la perspectiva de la investigadora. Además, brindar a los participantes la oportunidad de discutir y revisar los datos recolectados es un avance metodológico en la investigación cualitativa, y en este estudio, asociado a la ICA, se reveló como una posibilidad factible. 

## Conclusión

La Consulta de Enfermería realizada en la ESF con personas que tienen DM fue conformada por los(as) enfermeros(as) con elementos que se acercan al MACC, tales como el establecimiento de vínculos con los usuarios, la responsabilización, el cuidado longitudinal y el enfoque social y familiar. Sin embargo, se distanciaron de los supuestos de este modelo cuando hubo una comprensión limitada del modelo de atención, además de la postura profesional de culpabilización del usuario y de las debilidades en la aplicación del proceso de enfermería con las personas que tienen DM. Estos elementos que reflejaron la necesidad de cambio en la práctica asistencial fueron el punto de partida para sustentar el proceso educativo desarrollado con los(as) participantes por medio de la ICA.

La Investigación Convergente Asistencial se desarrolló durante un largo período y permitió promover un proceso de acción-reflexión-acción con resultados positivos para la translación del conocimiento, como: enfermeros sensibilizados y con voluntad de cambio, interesados en la profundización teórica y práctica del MACC y del PE, y colaborativos en la construcción e implementación de instrumentos y tecnologías que favorecieran el proceso de trabajo (protocolo y guía). Se observa, entonces, que las transformaciones ocurridas benefician a los usuarios y contribuyen a la cualificación de la asistencia de enfermería en la ESF. Así, las investigaciones que demuestran este compromiso de articular teoría y práctica son relevantes en el área de la Enfermería, ya que rompen con la idea utilitarista de la investigación y, de hecho, promueven cambios e introducen innovaciones en el sector de la salud. 

# Translation: Consulta de enfermagem e diabetes: processo educativo e transformador para os cuidados primários de saúde^*^

**DOI:** 10.1590/1518-8345.7546.4465

**Authors:** Silvana de Oliveira Silva, Andréa Carvalho Araújo Moreira, Alexa Pupiara Flores Coelho Centenaro, Nara Marilene Oliveira Girardon-Perlini, Teresinha Heck Weiller, Maria Denise Schimith

**Affiliations:** 1 Prefeitura Municipal de Santiago, Secretaria Municipal de Saúde, Santiago, Rio Grande do Sul, RS, Brazil.; 2 Universidade Federal de Santa Maria, Santa Maria, Rio Grande do Sul, RS, Brazil.; 3 Universidade Estadual Vale do Acaraú, Sobral, Ceará, CE, Brazil.; 4 Universidade Federal de Santa Maria, Palmeira das Missões, Rio Grande do Sul, RS, Brazil.

**Keywords:** Processo de Enfermagem, Diabetes Mellitus, Atenção Primária à Saúde, Modelo de Assistência à Saúde, Doença Crônica, Aprendizagem Baseada em Problema.

## Abstract

**Objetivo:**

analisar os elementos constitutivos que embasam a consulta de enfermagem com pessoas que têm diabetes *mellitus*, e desenvolver um processo educativo fundamentado nos pressupostos do Modelo de Atenção às Condições Crônicas e mediado por um processo reflexivo-dialógico com enfermeiros(as), visando à melhoria da qualidade da prática na Estratégia Saúde da Família.

**Método:**

pesquisa convergente assistencial realizada com 12 enfermeiros. Utilizou-se observação participante, entrevista individual semiestruturada e grupos de convergência para a coleta de dados, que foram tratados pela análise participativa, com abordagem interpretativa.

**Resultados:**

aproximaram-se do Modelo de Atenção proposto a promoção do autocuidado, o estabelecimento de vínculo profissional-usuário e o apoio a mudanças de hábitos das pessoas com diabetes. Por outro lado, atitudes, valores e conhecimentos que fragilizam o cuidado foram identificados como distantes. Constituíram-se pontos de convergência para a melhoria da qualidade da consulta o aprofundamento teórico e prático do modelo de atenção, diabetes, o enfrentamento da sobrecarga de trabalho, e a implantação de um guia e de um protocolo para o desenvolvimento da consulta de enfermagem. Constatou-se também o fortalecimento da autonomia dos enfermeiros, o despertar do pensamento crítico, e a busca pelo aperfeiçoamento e ressignificação da relação com o usuário.

**Conclusão:**

a consulta de enfermagem foi aprimorada a partir da participação ativa dos enfermeiros em um processo educativo, reflexivo e dialógico.

## Destaques

**(1)** A Consulta de Enfermagem às pessoas com DM pelos enfermeiros foram constituídas por elementos que se aproximam e se distanciam do MACC. **(2)** Aproximação com o MACC: o vínculo, a responsabilização, o cuidado longitudinal, e a abordagem social e familiar. **(3)** Distanciaram do MACC: compreensão limitada do modelo, postura profissional de culpabilização do usuário e das fragilidades do processo de enfermagem. **(4)** A consulta de enfermagem foi aprimorada por meio da participação ativa do enfermeiro em um processo educativo, reflexivo e dialógico.

## Introdução

O diabetes *mellitus* (DM) é uma doença crônica não transmissível que causou a morte de aproximadamente 2 milhões de pessoas em todo o mundo, em 2019. No período de 2000 a 2019, o DM registrou um aumento global de 3% no número de mortes, e, nas Américas, ultrapassou o acidente vascular cerebral para a medida de anos de vida ajustados por incapacidade, ocupando assim o segundo lugar^1^.

De acordo com a Federação Internacional de Diabetes (IDF), a prevalência da doença para países de baixa renda é de 5,5%^2^. No Brasil, identificou-se uma incidência de 8,6%, sendo que 68,2% da população tem conhecimento do seu diagnóstico, 92,2% dos que têm conhecimento do seu diagnóstico realizam tratamento medicamentoso, e desses, apenas 35,8% apresentam níveis de hemoglobina glicada controlados^3^. 

Assim, é fundamental ter a atenção direcionada aos danos atuais e futuros dessa doença, a fim de tencionar a implantação de políticas públicas eficazes, pois a maioria dos países pouco progrediu no cumprimento da meta 3.4 de Desenvolvimento Sustentável, que prevê uma redução de um terço na mortalidade prematura por doenças não transmissíveis entre 2015 e 2030. Nesse contexto, o rastreio e o tratamento do DM são intervenções de alta prioridade para reduzir a mortalidade por doenças crônicas^4^. 

A Atenção Primária à Saúde (APS) no Brasil, organizada em nível nacional por meio da Estratégia Saúde da Família (ESF) é a porta de entrada preferencial da pessoa com DM no sistema de saúde. É o ponto de atenção estratégico para melhor acolher as necessidades em saúde, incluindo o acompanhamento longitudinal e continuado^5^. Os tratamentos para DM baseiam-se em abordagens não farmacológicas e farmacológicas para o controle glicêmico, que devem ser escolhidas considerando os aspectos biopsicossocioespirituais de cada pessoa, mediante uma relação interpessoal entre profissional e paciente, porque esta influencia diretamente a adesão ao tratamento.

Destarte, o enfermeiro é o profissional de primeiro contato com o paciente, que deve assumir um papel de liderança na detecção, tratamento e reabilitação do diabetes. Cabe também a esse profissional apoiar os esforços de prevenção da doença e de promoção da saúde das pessoas que vivem com diabetes, permitindo-lhes assim participar na tomada de decisões relacionadas ao seu tratamento^6^. 

Para a Organização Mundial da Saúde (OMS), a contribuição da enfermagem para prevenir e gerir o diabetes no sistema de saúde é demonstrada por seis características: 1) coordenação dos cuidados para garantir que as necessidades de saúde do paciente sejam satisfeitas ao longo do tempo; 2) participação em uma abordagem multidisciplinar de cuidados baseada em uma relação integrada entre profissionais de saúde, permitindo que diferentes profissionais trabalhem em equipe para melhorar a qualidade dos cuidados; 3) mobilização e capacitação da força de trabalho de enfermagem para se especializar em doenças não transmissíveis, visando tratamentos mais eficazes e sustentáveis; 4) melhoria do acesso aos cuidados; 5) empoderamento de indivíduos e da comunidade; e 6) utilização da tecnologia para manter o acesso a serviços de saúde essenciais e reduzir a exposição à COVID-19^7^.

Há esforços de enfermeiros brasileiros em contribuir com a adesão ao tratamento do DM, proporcionando bases científicas para o cuidado de enfermagem, no intuito de direcionar ações que promovam maior adesão ao tratamento com a participação ativa do paciente, reduzindo complicações, hospitalizações e mortalidade^8^. Por outro lado, estudo internacional^9^ apontou que o diabetes ainda não é uma prioridade nos centros primários de saúde. Os enfermeiros explicitaram a importância de trabalhar a prevenção primária do diabetes, priorizando o cuidado das pessoas com pré-diabetes, e apontaram a necessidade do estabelecimento de diretrizes clínicas, apoio nos conhecimentos e habilidades assistenciais, além da implementação de um processo de trabalho interprofissional, aspectos cruciais na atenção à saúde da pessoa com diabetes que toma como fundamento o Modelo de Atenção às Condições Crônicas (MACC).

O MACC foi proposto por Eugênio Vilaça Mendes para atender às especificidades do sistema público de saúde brasileiro. Tem sua origem no Modelo de Cuidados Crônicos (MCC) advindo dos Estados Unidos, e incorpora outros dois modelos, o Modelo Pirâmide de Risco (MPR) e o Modelo da Determinação Social da Saúde, para se adaptar às exigências de um sistema de atenção à saúde público e universal como o Sistema Único de Saúde (SUS)^10^. Trata-se de um instrumento operacional, instituído no Plano Nacional de Enfrentamento das Condições Crônicas para o alcance dos objetivos de desenvolvimento do milênio até 2030.

A consulta de enfermagem (CE) orienta o pensamento crítico e o julgamento clínico do enfermeiro, direcionando-o para o cuidado à pessoa, família, coletividade e grupos especiais. No contexto da ESF e do cuidado às pessoas com condições crônicas, a CE favorece uma atenção resolutiva, proativa e humanizada, a partir de ações educativas que levam ao empoderamento dos usuários no alcance de metas estabelecidas no processo de cuidar e autocuidado.

Porém, são preocupantes as dificuldades enfrentadas na prática da CE no âmbito da APS, como: tempo escasso, falta de agilidade para o diagnóstico de enfermagem, problema na interface entre assistência e gerência, e demandas burocráticas excessivas^11^.

No campo científico, há muito para se investigar sobre a consulta de enfermagem com pessoa com diabetes no contexto da APS, dada a sua relevância, principalmente quando associada aos pressupostos do MACC. São poucos os estudos acerca dessa temática, que assume o compromisso de promover mudanças e/ou inovações na prática assistencial, adotando uma perspectiva constante de construção do pensar e do fazer.

Frente ao exposto, essa pesquisa tem como objetivo analisar os elementos constitutivos - conhecimentos, tecnologias, crenças e valores que embasam a consulta de enfermagem com pessoas que têm diabetes *mellitus*, e desenvolver um processo educativo, fundamentado nos pressupostos do Modelo de Atenção às Condições Crônicas e mediado por um processo reflexivo-dialógico com enfermeiros(as), visando à melhoria da qualidade da prática na Estratégia Saúde da Família.

## Método

### Tipo de estudo

Pesquisa convergente assistencial (PCA) que tem o intuito de, durante o percurso da pesquisa, promover mudanças e/ou inovações na prática assistencial, baseada na construção contínua do pensar e do fazer^12^. Sua elaboração atendeu às recomendações dos Critérios Consolidados para Relato de Pesquisa Qualitativa^13^. 

### Local do estudo

O estudo foi realizado em um município de médio porte localizado na região centro-oeste do estado do Rio Grande do Sul-Brasil, que integra o projeto de Planificação da Atenção à Saúde e tem 11 unidades de ESF, as quais cobrem 86% da população total.

### Período

A produção dos dados realizou-se no período de agosto de 2020 a dezembro de 2021, durante o qual a pandemia da COVID-19 assolava o país. Todos os cuidados de prevenção de transmissão foram tomados. 

### Participantes e critérios de seleção

Foram incluídos enfermeiros(as) que atuavam em ESF e com tempo de experiência em CE junto às pessoas com DM de no mínimo seis meses [conforme artigo 442-A da Consolidação das Leis do Trabalho (CLT), este é o tempo máximo de experiência que pode ser exigido por empregadores para fins de contratação]. Estabeleceu-se como critério de exclusão: enfermeiros(as) que por algum motivo estivessem afastados(as) do trabalho por mais de 30 dias durante o período de coleta de dados, o que não ocorreu. Deste modo, 12 enfermeiros(as) foram contatados por *e-mail* para reunião de apresentação da proposta e, em sua totalidade, consentiram em participar do estudo. 

### Coleta de dados

Adotou-se a triangulação de técnicas para a produção de dados: observação participante ativa^14^, entrevista semiestruturada^15^ e grupos de convergência^16^. 

O objetivo da observação participante foi captar e registrar os mais variados elementos e impressões sobre a condução da CE com pessoas com DM. Assim, utilizou-se um roteiro pré-estabelecido, que incluiu os seguintes aspectos: relação profissional com o usuário, comunicação, etapas do processo de enfermagem, registro de enfermagem, e ambiente.

A interação da pesquisadora no decurso das observações ocorreu de forma gradual. Durante a CE, buscou manter uma posição mais passiva, interagindo estritamente o necessário. A participação ativa ocorreu majoritariamente após a finalização da consulta, quando, entre um atendimento e outro, dialogava-se sobre o caso e compartilhava-se saberes a partir das percepções da pesquisadora e das dúvidas elencadas pelos participantes.

A interrupção das observações foi estabelecida mediante o critério de saturação teórica^17^, que consistiu na compilação individual, buscando identificar se os elementos propostos pelo roteiro foram explorados. Uma vez que todos os elementos foram contemplados, e nenhuma outra nova informação pertinente ao tema do estudo emergiu, encerraram-se as observações. Totalizando 120 horas, e com uma média de 10 horas/participante, os dados produzidos nesta etapa foram registrados em diário de campo e em gravador digital, imediatamente após o seu término, codificados e posteriormente transcritos para o editor de texto *Microsoft Word*. 

A entrevista individual semiestruturada foi realizada pela pesquisadora após o alcance da saturação das observações e antes dos grupos de convergência. Para tanto, foram incentivados a falar sobre o cotidiano das CE com pessoas com DM, abordando aspectos como objetivo, o que faz e como faz; sobre o MACC, seus conhecimentos e aplicabilidade no cotidiano; sobre os desafios e as potencialidades para realizar CE com pessoas com DM; e sugestões para os grupos de convergência. Um teste-piloto foi aplicado, porém não fez parte da amostra de participantes. Realizaram-se 12 entrevistas, agendadas previamente com os(as) participantes, nas unidades de saúde, audiogravadas, com duração média de 40 minutos. Em seguida, um auxiliar de pesquisa capacitado transcreveu o material no editor de texto *Microsoft Word*.

Os dados obtidos por meio destas duas técnicas subsidiaram o Grupo de Convergência (GC), que tem a finalidade de realizar a pesquisa em simultaneidade com a prática assistencial junto ao coletivo, com foco na educação em saúde ou na prática clínica. Neste espaço, busca-se promover discussões a respeito de determinado tema, foco da pesquisa, em um processo interativo entre participantes e pesquisador, de forma a permitir a dialogicidade^18^. 

Os encontros sucederam-se conforme cronograma elaborado com os(as) participantes, na sala de reuniões da Secretaria Municipal de Saúde, com tempo médio de 2 horas e 30 minutos. Foram audiogravados em gravador digital, e a pesquisadora contou com o auxílio de um assistente de pesquisa, discente do Curso de Mestrado em Enfermagem, que recebeu capacitação e contribuiu ativamente com o planejamento, a execução, a observação e a transcrição. 

Para o alcance da coesão, no grupo de convergência percorreu-se um processo de quatro fases, denominado o Processo dos Quatro Erres (R): reconhecimento, revelação, repartir e repensar^16^. A fase reconhecimento teve o intuito de possibilitar o primeiro encontro do grupo e estabelecer as relações entre participantes e pesquisadora de maneira que por meio do diálogo participativo ocorresse a coesão do grupo. Esta fase envolveu dois encontros. O primeiro teve como objetivo analisar os conhecimentos dos(as) participantes sobre a CE no que tange à sua finalidade e seus pressupostos. Para isso, foram realizadas atividades em grupo, incluindo a elaboração de cartazes, dramatização e debates. O segundo encontro concentrou-se nos conhecimentos sobre o MACC, com a utilização de palavras-chave e imagens como recursos para promover a interação e o diálogo.

A fase revelação compreendeu o momento em que os(as) participantes se identificaram com seus pares pela experiência em comum com a proposta dos encontros. Esse processo foi desencadeado quando foram incentivados a falar sobre suas vivências com a CE com pessoas com DM e elaborar os conceitos que regem sua prática assistencial. 

A fase repartir foi o momento em que o grupo, mediado pela troca de experiências, foi conduzido a tomar decisões compartilhadas com relação ao propósito do encontro. Esta fase teve três encontros e contou com uma participação mais ativa da pesquisadora. As atividades transcorreram com suporte de leitura, discussão de materiais científicos, dramatizações, estudo de casos e apoio matricial de nutricionista.

A fase repensar se caracterizou pela reflexão do grupo quanto às implicações dos problemas identificados e a possibilidade de transferir o que foi compartilhado e aprendido para o cotidiano dos(as) participantes^16^. Esta fase se deu em três momentos, com a utilização de estratégias como: técnica de *Brainstorm,* estudo de casos*,* dinâmicas de grupo e elaboração do guia para a CE com pessoas que têm DM.

Os encontros grupais foram planejados e desenvolvidos tomando como referência a Pedagogia Libertadora de Paulo Freire, a qual tem sido empregada em estudos nas áreas da educação, saúde e enfermagem. Nesse ponto, a Pesquisa Convergente Assistencial se articula com este referencial, uma vez que tem como alicerce o diálogo crítico libertador, baseado no princípio de que a reflexão crítica conduz à ação (prática), denominada práxis^19^. 

### Análise dos dados

A análise fundamentou-se na pesquisa participativa com abordagem interpretativa, realizada por meio da construção e devolutiva de narrativas^20^. Baseada nos pressupostos da Teoria Hermenêutica de Gadamer, essa abordagem considera que os atos de interpretação são dialógicos, explorando estratégias participativas e sugerindo algumas mudanças na forma clássica de conduzir as pesquisas. Dentre essas mudanças, duas estratégias são utilizadas: a construção de narrativa e validação, e a produção de consenso participativo^20^. Desta forma, o participante atua no processo de investigação, o que possibilita a ressignificação e a autorreflexão de suas condutas^21^. 

No decorrer do processo de transcrição e organização dos dados, foram realizadas diversas leituras em profundidade com o intuito de compreender e explorar novos depoimentos. Assim, foi possível perceber relatos reincidentes, peculiares, divergentes e contradições expressadas que envolviam a prática da CE, e realizar as primeiras inferências e interrogações. 

Diante dessas considerações, a análise foi executada da seguinte maneira: após a transcrição de cada uma das entrevistas semiestruturadas (n=12), foi construída uma narrativa. A seguir, a pesquisadora retornou ao campo e fez a leitura da narrativa para cada participante e, por meio do processo dialógico, o(a) participante refletiu sobre a narrativa e teve a oportunidade de contestar ou reforçar e validar. Com os grupos de convergência, do mesmo modo, foram produzidas nove narrativas, uma de cada encontro, que foram lidas e validadas com os(as) participantes na medida em que ocorriam os encontros. Sempre antes de iniciar o próximo grupo, reforçava-se a possibilidade de coprodução dos(as) participantes.

Como forma de facilitar a organização desses dados e a categorização dos núcleos argumentais, as notas de observação e as narrativas das entrevistas e dos grupos foram inseridas no *NVivo Software* Corporativo. Assim, com o desenvolvimento de 21 narrativas e as notas de observação, foi possível construir e encadear os núcleos argumentais, os quais buscaram responder à questão de pesquisa^20^.

### Aspectos éticos

A pesquisa seguiu as orientações da Resolução nº 466/12 do Conselho Nacional de Saúde^22^, além da Resolução nº 510/2016 do Ministério da Saúde, que enfatiza as especificidades éticas das pesquisas nas ciências humanas e sociais e de outras que utilizam metodologias próprias dessas áreas^23^. Foi aprovada pelo Comitê de Ética em Pesquisa da Universidade Federal de Santa Maria, sob parecer nº 4.209.003. Todos(as) os(as) participantes aceitaram participar do estudo e assinaram o Termo de Consentimento Livre e Esclarecido. O anonimato está garantido com os seguintes códigos: O (Observação) seguido da letra E (enfermeiro), numeral 1 a 12 e data (dia/mês/ano); NE (Narrativa Enfermeiro) seguido do numeral 1 a 12; NGC (Narrativa Grupo de Convergência) seguido do numeral 1 a 9. 

### Resultados

Participaram do estudo 12 enfermeiros, em sua maioria mulheres (n=11), com média de idade de 42 anos (mínima de 33 anos e máxima de 51 anos). O tempo médio de formação em Enfermagem foi de 12 anos (mínimo de 5 anos e máximo de 25 anos), e o tempo de atuação na ESF foi, em média, de 7,5 anos, sendo o menor tempo de 6 meses (n=1) e o maior de 11 anos (n=3).

Os resultados serão apresentados por meio dos seguintes núcleos argumentais: Elementos constitutivos da Consulta de Enfermagem com pessoas que têm DM que se aproximam e se distanciam dos pressupostos do Modelo de Atenção às Condições Crônicas; e Agora faz sentido: a convergência entre a pesquisa e o processo educativo.

### Elementos constitutivos da Consulta de Enfermagem com pessoas que têm DM que se aproximam e se distanciam dos pressupostos do MACC

Os elementos da CE com pessoas que têm diabetes na ESF abrangem características que se aproximam e se distanciam do MACC, conforme Figura 1.

**Figura 1:** Elementos constitutivos da Consulta de Enfermagem com pessoas que têm diabetes *mellitus*

*CE = Consulta de enfermagem; ^†^DM = Diabetes *mellitus*; ^‡^MACC = Modelo de Atenção às Condições Crônicas

Os(as) enfermeiros(as) enfrentam alguns desafios para realizar a CE às pessoas com diabetes que perduram há alguns anos, a saber: sobrecarga de trabalho, inexistência de protocolos de enfermagem e procedimentos operacionais padrão (POPs), e o quantitativo insuficiente de profissionais de enfermagem para suprir as demandas assistenciais e gerenciais. [...] *Acredito que o que mais limita a assistência ao usuário na consulta é a sobrecarga! Estou aqui para dar assistência ao usuário, só que eu não faço só isso! Tem as questões gerenciais do serviço, a demanda espontânea e administrativa* [...] (NE3). [...] *por várias vezes a enfermeira foi interrompida durante a consulta para resolver questões administrativas do serviço, como solicitar materiais, receber insumo, fazer contato com outro serviço, informar período de férias do agente comunitário; pediu licença e saiu da sala para avaliar lesão na sala de curativos* (NO 08/09/2020)*. Reconhecemos a necessidade de elaboração e implantação dos POPs e protocolos de enfermagem, porque presenciamos diariamente situações que, se tivéssemos esses instrumentos, teríamos mais autonomia* [...] *sem falar que maior qualidade na atenção aos pacientes. Assim nosso tempo seria melhor aproveitado* [...]. *Por esses motivos, procurando a questão da resolutividade na atenção primária, entendemos que uma das possibilidades são os protocolos, os POPs.* (NGC1). *Mesmo antes da pandemia o quantitativo de profissionais de enfermagem já era insuficiente. Já não estávamos dando conta porque na verdade seria necessária uma enfermeira para a parte assistencial e outra para a gerencial. E nós temos só dois técnicos de enfermagem na equipe, porém, seguidamente tem um que não está, porque ou está em férias, laudo, atestado, folga, ou outra atividade como agora, que está emprestado para outra unidade para dar suporte na vacinação.* (NE10).

Por outro lado, os(as) participantes reconheceram alguns fatores contextuais como meios potencializadores da consulta de enfermagem na ESF para pessoas com diabetes, como o apoio da gestão municipal, rede de atenção à saúde do município, e compartilhamento do cuidado com o ambulatório de especialidades; o reconhecimento da população do desempenho profissional dos enfermeiros; e o vínculo com os usuários e a relação profissional com o médico da unidade: [...] *me sinto contemplada nessa gestão. Nossas demandas são atendidas; temos horário protegido para reuniões de equipe e capacitações* (NE9). *O que potencializa a consulta é a atenção primária ser muito fortalecida no município.* [...] *Os usuários têm acesso, acolhimento, reconhecem a unidade como porta de entrada e nosso trabalho como enfermeiras* [...] (NE1). *A questão das interconsultas com a médica, pois auxilia na resolutividade* (NE4). [...] *temos o ambulatório especializado para encaminhar os usuários que necessitam de avaliação* [...] *e eles sempre nos dão retorno para implementar o cuidado* (NGC3). [...] *também dispomos de uma rede de atenção à saúde para os usuários aqui no município, como os laboratórios, centro de referência de assistência social, o núcleo de apoio à saúde da família, nutrição e tudo isso podemos disponibilizar para ele na Consulta, o usuário tem acesso sempre que precisa* (NE7).

### Agora faz sentido: a convergência entre a pesquisa e o processo educativo 

Da convergência entre a pesquisa e a prática assistencial emergiram as demandas para qualificar a Consulta de Enfermagem na ESF com pessoas que têm DM. O processo educativo desenvolvido no percurso dos grupos de convergência está representado na Figura 2.

**Figura 2:** Pontos de convergência entre pesquisa e prática assistencial

*CE = Consulta de enfermagem; ^†^MACC = Modelo de Atenção às Condições Crônicas; ^‡^DM = Diabetes *mellitus*

Neste estudo, a troca de conhecimentos entre pesquisadora e participantes subsidia ações concretas de mudanças e movimentos de reflexão-ação que resultaram na construção coletiva de um guia para a CE com pessoas que têm DM e a implantação de um protocolo de enfermagem. Tais mudanças estão representadas na Figura 3.

**Figura 3:** Transformações no cotidiano da Consulta de Enfermagem a partir do processo educativo-dialógico

Percepção da necessidade de aprofundamento teórico e prático da CE*	[...] *Precisamos aprofundar nossos conhecimentos em DM* ^*†*^ e cuidado com os usuários. Instituir um protocolo de enfermagem e um guia de CE* para nos auxiliar no dia-a-dia e estruturar melhor a CE*, todo o processo, coleta de dados e intervenções em referenciais teóricos (NGC^‡^7). [...] *Nas oficinas de planificação, a forma como o MACC* ^*§*^ foi apresentado, mais densa, o foco estava na organização da demanda do serviço e não no cuidado em si. Agora faz sentido para nós, conseguimos, com propriedade, dizer em que modelo de saúde atuamos, ou estamos procurando atuar. Agora está explícito qual é o norte (NGC^‡^4).
Elementos constitutivos da CE* fortalecidos	*Todas as ferramentas que vimos, a Sistematização da Assistência de Enfermagem, os diagnósticos de enfermagem, o uso das tecnologias, as próprias ferramentas que o sistema de informação oferece que não utilizávamos, não sabíamos da importância e o quanto qualifica a CE* * [...] *os saberes científicos em relação às definições de enfermagem, pessoa, ambiente, cuidado, saúde, sobre a CIPE* ^*||*^ que não sabíamos como utilizar, elaborar um diagnóstico. Conhecer e discutir sobre tudo isso e os modelos de atenção à saúde foi essencial nesse momento de mudança **(NGC** ^‡^ **9).**
Inquietações e desejos para a mudança da prática	*Nos vimos em muitos exemplos citados e percebemos que é preciso mudar! Desconstruir os próprios pré-conceitos para transformar nossa prática. Ainda não nos sentimos preparadas, mas percebemos a necessidade de considerar o que a pessoa traz para definir metas com ela* **(NGC** ^‡^ **6).**
Elementos constitutivos da CE* ressignificados	*Agora tudo faz sentido! A consulta é com as pessoas que convivem com diabetes. Tivemos que repensar isso, sobre o contexto em que está inserida e nosso papel profissional. A relação com o usuário que a gente não considerava muito! Mas considerar ele como parte do processo de cuidado com diabetes é muito importante. Isso foi ressignificado. Porque a gente achava que o considerava, mas na prática, nossas ações eram prescritivas. Não considerávamos o usuário como ator do processo, suas reais necessidades, a perspectiva dele como o agente de mudança. A nossa prática era assim e foi ressignificada. O trabalho em equipe. Falamos muito sobre isso, “Não é saudável centralizar tudo em nós, isso que causa a sobrecarga”, e foi possível ressignificar a importância de trabalhar em equipe* **[...] (NGC** ^‡^ **9).**
Possibilidades de minimizar os desafios	*Fazer sem planejar tem sido nossa prática, e percebemos que é necessário instituir a divisão de tarefas, planejar em curto espaço de tempo, analisar a situação. E o dividir, delegar atribuições, irá fazer toda a diferença para alcançar um resultado muito bom e não causar a sobrecarga de trabalho que tanto sentimos. Quanto à comunicação, concluímos que para nós pode estar claro o que queremos, mas para a equipe e o usuário talvez não, e atentar para isso é fundamental* **(NGC** ^‡^ **5).**
Transformações na prática da CE* com as pessoas que têm DM^†^	*Nossos encontros têm ajudado bastante! Temos procurado colocar em prática, e a consulta ficou bem melhor! Os usuários se sentem valorizados com as mudanças que temos inserido. A CE* * *tem outro teor, parece que ficou mais grandiosa para eles* **[...]** *. Ter revisitado o conhecimento sobre a alimentação foi fundamental. Um cuidado que muitas vezes nem nós temos, e aqui, um outro desafio, não só a alimentação da pessoa com DM* ^*†*^ , mas uma alimentação saudável para toda a família! **(NGC** ^‡^ **7).** *Depois que passamos a delegar algumas funções para a equipe, percebemos que foi possível nos dedicar mais à CE* * *. Eu sinto que melhorou* **(NGC** ^‡^ **9).**

*CE = Consulta de enfermagem; ^†^DM = Diabetes *mellitus*; ^‡^NGC = Narrativa grupo de convergência; ^§^MACC = Modelo de Atenção às Condições Crônicas; ^||^CIPE = Classificação internacional das práticas de enfermagem

## Discussão

As pessoas que vivenciam uma condição crônica como o diabetes requerem um cuidado adequado já no início do diagnóstico, para que experimentem um processo de adaptação favorável à sua nova condição de saúde e mantenham o equilíbrio de seu bem-estar^24^. Por isso, vários modelos organizacionais são utilizados com o objetivo de educar e apoiar a autogestão de pessoas com doenças crônicas, sendo o Modelo de Cuidados Crônicos (MCC) o mais utilizado, conforme destacado em um estudo de revisão^25^. 

É importante mencionar que o MACC tem seus fundamentos embasados no MCC e, para além de um modelo organizacional, ele incita mudanças de paradigmas na assistência, com vistas à integralidade da atenção, sendo o usuário o centro do cuidado^10^. Nesta pesquisa, evidenciaram-se elementos da CE com pessoas com DM que se distanciam dos pressupostos do MACC, na medida em que se observa uma visão hegemônica da relação enfermeiro-paciente, com práticas centradas na doença e curativistas. Pesquisa internacional realizada recentemente explorou as percepções de enfermeiros sobre o cuidado centrado na família de pessoas adultas com diabetes e, de forma semelhante, o modelo médico dominante ainda é um desafio a ser superado^26^.

Frente a essa realidade, os enfermeiros brasileiros devem refletir sobre a importância do seu papel ético e político no cuidado às pessoas com doenças crônicas, no cumprimento e fortalecimento dos princípios do Sistema Único de Saúde (SUS), assim como na visibilidade da profissão. O modo como os(as) participantes deste estudo compreendem e operacionalizam o MACC se diferencia exponencialmente de outras realidades, a exemplo da Áustria, onde os enfermeiros são reconhecidos pela sua competência e detêm preferência dos usuários para liderar programas/modelos de cuidados crônicos. Isso tem implicações práticas na forma como os modelos de cuidado são dotados de profissionais, e apresenta uma oportunidade para a força de trabalho da enfermagem desempenhar um papel de liderança na prestação de serviços^27^. 

Em Singapura, os usuários em acompanhamento para diabetes no sistema de cuidados primários em saúde que adotam o MCC também relataram satisfação com os cuidados de enfermagem prestados, com a continuidade dos cuidados, e com a abordagem centrada no paciente, envolvendo o estabelecimento de metas e a resolução de problemas^28^.

Contudo, os(as) enfermeiros(as) neste estudo identificaram vários desafios para realizar a CE com pessoas que têm DM, relacionados com o processo de trabalho, como sobrecarga, atribuições gerenciais e assistenciais sobrepostas e a necessidade de implantação de protocolos. Para superar tais desafios, enfermeiros bolivianos apontaram necessidades de apoio no cuidado de pessoas com diabetes em nível primário, englobando quatro dimensões: organização dos cuidados e políticas de saúde; reforço nos conhecimentos, habilidades e atitudes da equipe de saúde; capacitação das pessoas que vivem com diabetes e suas famílias; e educação sobre diabetes em nível comunitário^29^.

Os desafios apontados nesta pesquisa poderão interferir negativamente na qualidade da assistência prestada, e por consequência acarretar dificuldade para o controle metabólico e complicações como o pé diabético e amputações de membros das pessoas com diabetes. Assim, é necessário um gerenciamento multidisciplinar eficaz da condição clínica do diabetes, o que requer a contratação adequada de profissionais de saúde, uma equipe bem definida, adesão a diretrizes baseadas em evidências, uma boa infraestrutura para a coleta de dados e mecanismos para a melhoria de qualidade com base na revisão de dados clínicos^30^. 

Nesta pesquisa, o método PCA possibilitou aos(às) enfermeiros(as) refletir sobre sua prática na CE com pessoas que têm DM e iniciar um processo de mudança. Desta maneira, o reconhecimento por parte dos(as) enfermeiros(as) da necessidade de superar desafios se deu mediante um processo educativo que estimulou a reflexão e a criticidade, tornando-os agentes ativos e transformadores da realidade, o que contribuiu para o estabelecimento de estratégias para minimizá-los. De forma semelhante, estudos realizados com o mesmo método em outras áreas assistenciais também possibilitaram investimento na formação em serviço, visando à qualificação do cuidado de enfermagem^31-32^. 

Os(as) participantes da pesquisa ressignificaram a prática da CE com pessoas que têm DM, compreendendo o exercício do(a) enfermeiro(a) como a arte de perceber continuamente e de forma minuciosa as necessidades do ser cuidado, valorizando os aspectos subjetivos, que o permitirá desvelar a pessoa em sua integralidade, resultando na escolha da melhor intervenção para ela^18^. 

A postura profissional foi percebida como uma necessidade de mudança, sendo ressignificada para um cuidado corresponsável, que os(as) participantes denominaram de “cuidar com o paciente”. Evidências científicas apontam que a relação profissional-usuário centrada, com postura dialógica, que prima pela emancipação do sujeito e por aspectos biopsicossociais para determinar as necessidades de cuidado, potencializa o cuidado com as pessoas que têm DM^33-34^ ^35^. 

A percepção da necessidade de formação e qualificação profissional foi fundamental. A educação permanente em saúde é parte integrante do processo de trabalho e permite o desenvolvimento de competências e habilidades coerentes com o modelo de atenção preconizado pelo SUS^36^. Ademais, quando os enfermeiros recebem treinamento e suporte estruturado, e utilizam algoritmos de tratamento e tecnologias, o cuidado de enfermagem torna-se mais eficaz, impactando nos valores de hemoglobina glicada, com reduções de 0,03 a 2%^37^. 

As demandas por aprofundamento teórico do MACC e CE levantadas pelos(as) participantes foram conduzidas de forma a garantir uma aprendizagem significativa baseada na realidade^38^, gerando satisfação com a metodologia utilizada. Esse tipo de aprendizagem possibilita a melhoria do pensamento crítico, da autonomia, da motivação para aprender, da atitude ativa, da capacidade de trabalhar em equipe e de resolver problemas. Para tanto, concorda-se com a prerrogativa de que os enfermeiros, durante seu processo de formação permanente, podem desenvolver competências para atuar de forma efetiva na APS, refletindo na e sobre a prática e decidindo sobre a necessidade de permanecer em constante formação para lidar com as questões advindas dessa prática^39^. 

Vale destacar a importância dada pelos(as) participantes para as discussões sobre o saber científico da enfermagem, retomando diferenças conceituais de sistematização da assistência de enfermagem e o processo de enfermagem (PE), assim como a utilização de sistemas de linguagens padronizadas, ou seja, saberes e ferramentas que qualificam a CE com pessoas que têm DM. No ano de 2024, foi publicada a resolução do Conselho Federal de Enfermagem (COFEN) nº 736, que, em seu Artigo 2^o^, conceitua e qualifica o PE como um método que orienta o pensamento crítico e o julgamento clínico do enfermeiro para o cuidado, que deve estar fundamentado em suporte teórico, como modelos de cuidado, sistemas de linguagens padronizadas, instrumentos de avaliação de predição de risco validados e protocolos^40^. Deste modo, pode-se inferir que o referido conceito se articula fortemente ao experienciado pelos enfermeiros no decurso do processo educativo-reflexivo. 

Neste estudo, foi primordial obter o resultado de que os(as) participantes perceberam que o PE tem como objetivo interferir positivamente na assistência de enfermagem a pessoas que têm DM, pois o direciona ao raciocínio clínico e à prescrição de cuidados de enfermagem, visando a uma maior qualidade da assistência. Conforme evidenciado em estudo de revisão sistemática, faz-se necessário o desenvolvimento de mais pesquisas que analisem a utilização do processo de enfermagem em contextos de saúde comunitária e saúde pública e, da mesma forma, que avaliem as intervenções de enfermagem e a satisfação da população com o PE^41^.

Outro resultado aponta para a revisão da gestão do trabalho, com um desejo manifestado pelos(as) participantes de adotar uma prática de colaboração interprofissional, quando citaram algumas das suas características, como o trabalho em equipe, a comunicação, a divisão de tarefas e o planejamento. Enfermeiros da APS da Noruega reconheceram que aprender as habilidades de colaboração interprofissional representa uma oportunidade significativa de melhoria da qualidade nos serviços de APS^42^.

Nesse processo sistemático de construção e reconstrução da práxis ocorreram desdobramentos no contexto dos(as) enfermeiros(as), como a construção do guia para o desenvolvimento da CE junto às pessoas com DM, o qual posteriormente foi validado^43^, e a implantação dos protocolos de enfermagem do Conselho Regional de Enfermagem do Rio Grande do Sul (Coren/RS), por meio do Decreto Municipal nº 067/2023. Dessa maneira, apoia-se vigorosamente *designs* de estudos participativos, pela possibilidade de identificar soluções inovadoras com potencial para melhorar o tratamento centrado na pessoa com diabetes^44^. Nessa perspectiva, é fundamentalmente importante na construção de tecnologias educativas favorecer um alto nível de envolvimento das pessoas com diabetes como parte igualmente interessada^45^.

Portanto, verifica-se nesta pesquisa alguns pontos fortes que se assemelham a estudo anterior que também utilizou a PCA^46^, como o fato de considerar a realidade e a experiência dos(as) participantes em seu local de trabalho, o que favorece o conteúdo e a sua exequibilidade, exprimindo a potencialidade da translação do conhecimento para a prática. 

Nesse sentido, a transformação da prática gerou satisfação direta ao usuário, e consequentemente valorização profissional. Portanto, ratifica-se que nos espaços de encontro entre as pessoas, as barreiras hierárquicas implicadas na lógica biomédica são rompidas, há a democratização do saber em saúde, a ressignificação ao longo de toda a trajetória e um constante movimento de ação-reflexão-ação^47^. Acredita-se que as estratégias utilizadas nesta pesquisa que culminaram em transformações positivas no processo de trabalho dos(as) enfermeiros(as) da ESF possam ser adaptadas e aplicadas em outras realidades.

As limitações dessa pesquisa são creditadas a dois fatores. Não foi prevista, no desenho da pesquisa, a participação de usuários(as), o que poderia ter triangulado o resultado trazido pelos(as) enfermeiros(as). A pesquisa desenvolvida, mesmo sendo uma PCA, não prevê a continuidade do monitoramento da CE para acompanhar a melhoria da qualidade atingida.

A escolha da análise participativa para a PCA é um avanço do conhecimento científico da enfermagem, pois possibilitou que os(as) enfermeiros(as) usassem a lente da pesquisadora. Além disso, permitir que participantes discutam e revisem os dados coletados é um avanço metodológico na pesquisa qualitativa, e neste estudo, associado à PCA, revelou-se uma possibilidade factível. 

## Conclusão

A Consulta de Enfermagem realizada na ESF com pessoas que têm DM foi constituída pelos(as) enfermeiros(as) por elementos que se aproximam do MACC, tais como o estabelecimento de vínculo junto aos usuários, a responsabilização, o cuidado longitudinal, e a abordagem social e familiar. Entretanto, distanciaram-se dos pressupostos deste modelo quando houve uma compreensão limitada do modelo de atenção, além da postura profissional de culpabilização do usuário e das fragilidades na aplicação do processo de enfermagem com as pessoas que têm DM. Esses elementos que retrataram a necessidade de mudança na prática assistencial foram o ponto de partida para subsidiar o processo educativo desenvolvido junto aos(às) participantes por meio da PCA.

A Pesquisa Convergente Assistencial se desenvolveu por um longo tempo, e permitiu promover um processo de ação-reflexão-ação com resultados positivos para a translação do conhecimento, como: enfermeiros sensibilizados e com vontade de mudança, interessados no aprofundamento teórico e prático do MACC e do PE, e colaborativos para a construção e a implantação de instrumentos e tecnologias que favorecessem o processo de trabalho (protocolo e guia). Percebe-se, então, que as transformações ocorridas beneficiam os usuários e contribuem para a qualificação da assistência de enfermagem na ESF. Assim, pesquisas que mostram esse comprometimento em articular teoria e prática são relevantes na área da Enfermagem, pois rompem com a ideia utilitarista de pesquisa e, de fato, promovem mudanças e trazem inovações para o setor da saúde.
